# Optoelectronic nose based on an origami paper sensor for selective detection of pesticide aerosols

**DOI:** 10.1038/s41598-020-74509-8

**Published:** 2020-10-14

**Authors:** Mohammad Mahdi Bordbar, Tien-Anh Nguyen, Anh Quang Tran, Hasan Bagheri

**Affiliations:** 1 Chemical Injuries Research Center, Systems Biology and Poisonings Institute, Baqiyatallah University of Medical Sciences, Tehran, Iran; 2 Department of Physics, Le Quy Don Technical University, Ha Noi, Viet Nam; 3 Department of Biomedical Engineering, Le Quy Don Technical University, Ha Noi, Viet Nam

**Keywords:** Chemistry, Analytical chemistry, Sensors

## Abstract

This study introduces an applicable colorimetric sensor array for the detection of pesticides in the vapor phase. The array consisted of six metal nanoparticles spotted on the piece of filter paper. 3D-origami pattern was used for the fabrication of a paper-based sensor to decrease the effect of the nanoparticles leaching after exposure to analytes. Exposure to pesticide aerosols caused changes in the color of the array due to the aggregation of nanoparticles. These changes provided selective responses to thion pesticides such as malathion, parathion, chlorpyrifos, and diazinon. The sensing assay could also differentiate between aliphatic and aromatic thions and discriminate amine-containing compounds from the other studied analytes. These finding results are clearly confirmed by both visual detection and multivariate statistical methods. The proposed sensor was successfully developed for the quantitative measurement of pesticide aerosols at a very low concentration. The limit of detection of this method determined for malathion, parathion, chlorpyrifos and diazinon were 58.0, 103.0, 81.0 and 117.0, respectively. Moreover, the array could be employed to simultaneously analyze four studied pesticides. The statistcal results confirmed that the method has high performance for concurrent detection of thions as a major air pollutant without the interference of other species.

## Introduction

Air pollution occurs due to the propagation of toxic materials produced by human activity or natural disasters. It was reported that the air pollution can be harmful to more than seven millions of people in the world and other living organisms every year^1^. Different substances such as carbon monoxide, sulfur dioxide, nitrous oxides, methane and pesticides spread to the environment are considered as air pollutants^2^. Although pesticides are used to repel agricultural pests, they are resistant to physicochemical conditions and remain in the farm environment for long time^3^. Pesticides can be distributed in the form of aerosol mist in the air streams. Thus, they possibly enter to bodies of farmers and animals through respiration and disrupt the immune system by inhibiting the activity of acetylcholinesterase enzyme (AChE)^4^. Pesticide contamination has devastating effects on the function of the lung, cardiovascular, and nervous systems; in the most acute cases, it can lead to death^4^. Therefore, the amount of pesticides in the air should be constantly monitored and processed.

It is generally preferred to use gas chromatography coupled with a mass spectrophotometer^5,6^ or enzyme-based electrochemical methods^7–9^ to analyze pesticide pollution, which are suffering from the high cost of materials and instruments. These detection methods are also complexity in the detection procedure and needing standard working conditions or professional operators. Although some of these mentioned drawbacks have been solved by using advanced biosensor devices, these methods still tolerate two important difficulties: the choice of an appropriate adsorbent for the sampling of pesticides in the gaseous phase and finding a suitable extraction method to recoverthe pesticide compounds from the ambient air matrix^10^. The preparation procedures are also commonly time consuming and the complicated measurement. Moreover, the sampling and extraction steps can negatively affect the validity, accuracy and precision of analytical assays if improper methods are selected.

An Electronic nose (E-nose) is a packing set of gas sensors, which facilitates the identification process by assembling all experimental procedures, such as sampling and detection, in a simple device^11^. Since Persaud and Dodd introduced the first E-nose to differentiate between wide types of odors^12^, different gas sensor arrays have been developed for evaluating the environmental quality^13,14^. These devices have been fabricated by assembling some types of metal oxide semiconductors^15^, conducting polymer^16^, graphene^17^, or carbon-based nanoparticles^18^, with weak interactions with analyte through physical adsorption or van der Waals interactions^19^. However, the environment humidity can strongly influence the response of these sensor types^19^.

The color-based E-nose invented by Suslick could be a good alternative to previous methods, in which the covalent interactions occur between the receptors and the targets^20^. These sensor types are produced by immobilizing the organic dyes or inorganic complexes on the inert substrate such as silica gel plate and polymeric paper (e.g., polyvinylidene fluoride)^20^. In terms of structure, the design of E-nose has some advantages, such as simplicity, cost-effectiveness, and portability^21^. Utilizing this format, Chulvi et al. report a chromogenic sensor array created by 16 organic dyes for discriminating of 9 nerve agents or organophosphates^22^.

Dyes, as sensing elements, show high potential to detect a variety of essential materials, such as food, beverage, toxic gas, bacteria and diseases^20^. However, they still have drawbacks in the sensitivity and sometimes in selectivity. To overcome these issues, nanoparticles (NPs) can be used due to their unique physicochemical properties, such as surface plasmon resonance, which make them more capable of determining trace amounts of analytes^23^. Another advantage of NPs is that the chemical components of NPs (types of metal cores and stabilizing agents) can affect their selective responses to certain targets^24,25^.

Although the previous color based E-noses (Opto-E-nose) show considerable capabilities, the fabrication steps are commonlyexpensive and complicated. Moreover, The brittle polymer substrates and sensing elements of the sensing platform are required to be mixed with a stabilizer for preventing the leaching of the sensing materials when exposing to analyte vapors^20^. Recently, filter paper has been used as an alternative substrate because of its interesting features such as availability, cheapness, flexibility and biocompatibility^26–29^. The analytical devices based on a filter paper substrate are developed by using different techniques^30^. Among them, the ink-printing method is a popular and accessible procedure, creates hydrophobic barriers on the paper surface and separate the sensory areas from each other^31^. Also, the device can be constructed in different patterns like microfluidic channels, lateral flow, dipstick and origami form^32^. For detection of volatile compounds, the origami-based paper sensors show their promising advantages, which allow vapor to be absorbed in the paper pores and then direct detected. The origami template can also prevent the leaching of the sensing elements or the coffee effect after interacting with analytes, which allows maintaining the fast responses of the sensing assay^30^.

In this study, we utilize the advantages of origami papers and the novel physiochemical properties of NPs for developing a new sensing array to identify pesticide vapors. Unlike previous E-noses that focused on detecting nerve agent compounds, this study aims to estimate the percentage of pollution caused by pesticides in the agricultural environment. Since sulfur-containing organophosphates are commonly used in the farms, the materials such as malathion, parathion, chlorpyrifos and diazinon are selected for analysis.Importantly, none-enzymatic and high sensitive sensing elements based on metallic NPs are employed to design an affordable sensing device. In this fabrication approach, NPs are functionalized by inexpensive capping agents (cysteamine, tyrosine and tannic acid), with various reactive sites such as amino, thiol, hydroxyl and carboxyl groups. It allows NPs to directly interact with different studied analytes. The effective interactions between the sensing array and target molecules are obtained at optimal conditions, which result in the unique response of the sensor for each analyte. The readout method can be not only simply observed by naked eyes, but also comprehensively investigated by image processing tools. Our proposed method shows a simple and efficient assay for both qualitative and quantitative analyses of studied pesticides in the vapor phase.

## Results and discussion

Gold and silver nanoparticles, compared to other NPs, have attractive physicochemical properties and excellent capabilities for colorimetric measurements^33^. Further, unlike the other elements of sensors, these NPs have a good sensitivity that allows quantifying the traces of studied targets because of their higher absorption coefficient^34^. Arranging nanoparticles in the array structure should cause a selective pattern for each analyte. Therefore, NPs should be prepared by capping agents, which are different in size, surface electrical charge, and active site. For instance, in the proposed array, cysteamine is the small size of capping agents, thus expecting all pesticides to interact with these NPs easily. However, tannic acid is a huge molecule, and its NPs cannot be linked to some organophosphate due to steric hindrance. Also, the surface of NPs are covered by either positive or negative electrical charges. As an example, the positive electrical charges are shown for NPs synthesized by cysteamine and tannic acid; however, the surface of tyrosine-modified NPs are negative. Meanwhile, the Cys-capped NPs are more positive than TA-functionalized NPs. Therefore, the electrostatic interactions occur between Cys-capped NPs and more negative analytes while Tyr-capped NPs react with more positive pesticides. The variety of functional groups in the chemical structure of stabilizing agents causes the NPs to participate in different H-bonding, nucleophilic, or electrophilic interactions. For example, the active sites of Tyr-AgNPs are carboxyl and amino groups; however, AuNPs with the same capping agent enter reactions through carboxyl and carbonyl substituents^35^. Generally, it seems that the array fabricated by diverse NPs results in specific responses to a typical analyte. The following statements represent the efficiency of the paper-based sensor array prepared by NPs in details:

### Evaluation of sensor fabrication process

Field emission scanning electron microscopy (FE-SEM) was employed to confirm that synthesized NPs coated the paper. Fig. S1a indicates six individual spots on the paper surface. By investigating the morphology of each spot (Fig. S1b–g), it was found that the NPs were homogeneously distributed on the specified hydrophilic area. The distribution of NPs on the surface of paper was confirmed by SEM mapping which are shown in Fig. S2. The additional information could be obtained by SEM associated with the energy dispersed spectroscopy (EDX) used for elemental analysis of each NPs. As seen in Fig. S3, two strong peaks were observed at 3.0 keV and 2.3 keV in the EDX spectra of Cys-AgNPs and Cys-AuNPs, approving the formation of NPs and their immobilization on the paper substrate. The density of the main element in each NP structure was calculated by EDX analysis; the results are represented in Table S1.

The repeatability of the procedure for injecting the NPs on the surface of detection spots was examined by the preparation of five individual sensor arrays through the same process. For each sensor, the RGB values of each sensing element were determined, as presented in Table S2. Five individual numerical values were obtained for each color element (red, green, and blue). The relative standard deviation (RSD) of these data was calculated. As observed in Table S2, the amount of RSD % was lower than 2% for each measurement. Therefore, there was no systematic difference between the color intensities obtained for each sensing element. As a result, a high repeatable process was used to make the proposed Opto-E-nose.

To find the tolerance of the sensor against the ambient humidity, the relative humidity of the test box environment changed from 0 to 100%, and the response of the sensor were monitored. As shown in Fig. S4a, no significant changes were observed in the color of NPs when the humidity increased from 0 to 50%. At higher values, NPs aggregated or washed away from the paper surface.

Moreover, the durability of the sensors was checked by other experiment. Here, several sensors were fabricated and packed into a plastic packet for a certain period. The color of each sensing element was determined weekly. As illustrated in Fig. S4b, the color of sensors did not change for three weeks. By increasing the storage time, NPs aggregated due to changes in the physical conditions of the environment.

### Optimized condition

In order to experience an analytical assay with good sensitivity and selectivity, parameters with significant effects on the response of the sensor should be optimized. Since this study aims to discrimination and determination of pesticides, optimization should be individually performed for all sensing elements. Each element of the sensor comprises buffer and NPs; hence, it seems that the amount of NPs, type, concentration and pH of the buffer solution, play key roles in achieving suitable results. The optimal value is obtained by discrimination ability function (DAF) ^36^ defined as the following equation:

$$ DAF = \frac{n\mathop \sum \nolimits_{i} \left( {\overline{X}_{i} - {\mathop{X}\limits_{}^{=}}}\right)^{2} }{{\mathop \sum \nolimits_{i} \mathop \sum \nolimits_{j} \left( {X_{ij} - \overline{X}_{i} } \right)^{2} }} $$

The numerator of the fraction explains the between-analyte variations, and the denominator of the fraction introduces the within-analyte variations. In this equation, the symbols indicate the total number of analytes (n), the *j*th is the determination of *i*th analyte (*X*_*ij*_), the mean of five repetitive measurements of *i*_*th*_ analyte $(\overline{X}_{i} )$, and the total average of assay responses obtained by all studied analytes $({\mathop{X}\limits_{}^{=}}).$ For each parameter, the experiment with high values of DAF shows the optimal condition. The concentration of each analyte for optimization experiments was 450.0 ng mL^−1^.

In order to find the appropriate volume of NPs for preparing sensing elements, 0.5 µL of buffer was mixed with different amounts of NPs in the range of 0.1–0.5 µL. As seen in Fig. S5, the response of DAF was improved by increasing the volume of NPs up to 0.4 µL. The higher volumes negatively affected the DAF responses since the color of the NPs masked the considerable changes due to the interactions. Therefore, each sensing element was made by mixing 0.5 µL of the buffer, 0.4 µL of NPs, and 0.1 µL of deionized water.

The performance of the assay depends on the pH of the environment. Thus, the optimal pH influenced the robustness of electrostatic or H-bonding interactions between analyte and sensors^33^. To investigate the pH effect, the sensor response was separately monitored in the media with certain pH values in the range of 3.0–11.0. As observed in Fig. S6, the sensor showed a high potential for detecting the analytes in the alkaline media with a pH value of 9.0. The unfavorable responses were obtained at other pH values. Probably, at lower pH, the reaction sites of capping agents were blocked with hydronium ions^32^. On the other hand, at higher pH, the interaction of NPs and pesticides was disturbed in the presence of alkaline metal or hydroxyl ions as interfering species^32^. As a result, pH 9.0 was used for further studies.

Later, the experiment was performed in two different types of buffer: borate and Tris. As illustrated in Fig. S7, the desirable results were achieved using the borate buffer. For further studies, NPs were mixed with this buffer to create sensing elements.

To evaluate the ionic strength of the media, the concentration of buffer solution was varied from 0.05 to 0.2 M. As seen in Fig. S8, the appropriate response was observed in the buffer solution with the concentration of 0.1 M. The higher concentrations had an unsuitable effect on the desired interaction, and the response of DAF decreased constantly. Therefore, the buffer concentration was adjusted at 0.1 M for further studies.

The incubation between sensing elements and studied pesticides was investigated in a period. In this duration, the sample was sprayed into the test box, penetrated the paper, and interacted completely with NPs. The time required to perform all these steps for each analyte is shown in Fig. S9. As clarified, the response time of the sensor was 15 min for chlorpyrifos and diazinon and 20 min for malathion and parathion. By increasing the time of interaction, the color of the sensor did not change, and the response of the array was constant. Since pesticides should be determined at the same condition, the image of the sensor was recorded after 20 min.

### Colorimetric responses

**Figure 1:**
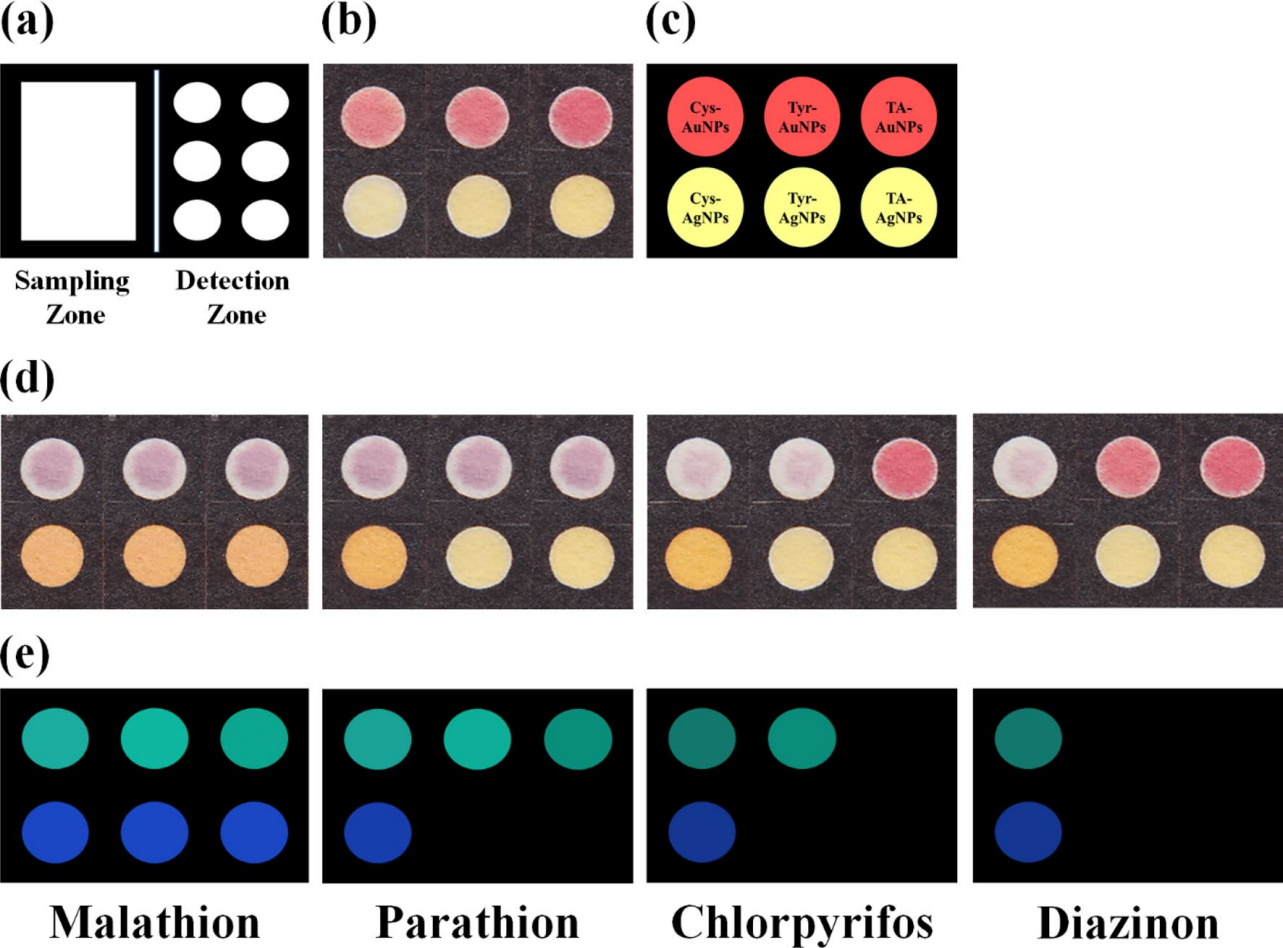
(**a**) The proposed pattern of paper based E-nose, (**b**) a typical fabricated sensor array, (**c**) Introduction of NPs used in the structure of sensor. (**d**) The colorimetric response of sensor array and (**e**) colorimetric difference maps for four studied pesticides. The concentration of pesticides used in this study was 450.0 ng mL^−1^. Each sensing element was prepared by mixing 0.5 µL of borate buffer (0.1 M) with 0.4 µL of a certain NPs and 0.1 µL of deionized water. The pH of mixture was adjusted at 9.0.

Figure 1 shows the desired pattern for the sensor array fabrication. The paper-based E-nose was individually exposed to aerosols of four pesticides at the optimal conditions. The responses of the assay to each analyte are shown in Fig. 1d. As can be observed, the color of sensing elements changed after exposure to the analyte due to the NPs aggregation. The presence of a pesticide reduced the electron repulsion or created a bridge between two NPs, leading to a decrease in the distance of NPs^37^. Based on this event, the yellow color of AgNPs turned to orange or brown, and the color of AuNPs changed from red to pale or intense purple. As seen, all NPs aggregated in the presence of malathion. This pesticide is an aliphatic compound with a range of active sites, including carbonyl, P=S, thiol groups, besides free paired electrons of oxygen. Thus, this material had a strong H-bonding and nucleophilic interactions with NPs. The small size of this chemical allowed it to participate in the interaction with TA-capped NPs. Parathion pesticide had a high tendency to AuNPs due to the high affinity of gold to sulfur atoms. However, it interacted with only Cys-capped AgNPs. The interaction of parathion and NPs generates a complete resonance in the aromatic ring bonded to nitro groups, forming a stable complex between analyte and NPs. Possibly, electrical repulsion and steric hindrance prevented the interaction of this pesticide with AgNPs synthesized by Tyr and TA, respectively. The other aromatic pesticides, diazinon, changed only the color of NPs prepared by cysteamine. Diazinon with some reaction sites, such as P=S, amino, and oxygen groups, had a part in covalent, nucleophilic, and H-bonding interaction with NPs. The positive charges on the surface of NPs caused the electrostatic interaction between NPs and analytes. The NPs synthesized by TA and Tyr could not show any response to diazinon because of the interference methyl groups in its chemical structure. Possibly, Tyr-capped NPs did not accumulate in the presence of diazinon due to both electrostatic and amine–amine repulsion. This happened for Tyr-AgNPs when exposing to chlorpyrifos. Similar to diazinon, chlorpyrifos resulted in aggregation of Cys-modified NPs. It could also affect the Tyr-AuNPs and change the color of these sensing elements. In the chlorpyrifos, chlorine substituents were replaced with methyl groups, and spatial disruptions decreased; thus, it could penetrate the space of AuNPs and create a complex with them. The aggregation of Cys capped AgNPs and Cys modified AuNPs in the presence of malathion were investigated by FE-SEM and the respective images were represented in Fig. S1h and Fig. S1i.

Color profiles (Fig. 1e) indicates difference between the color of sensing elements before and after exposure to analytes. Evidently, the visible observations were exactly in line with the results obtained by the image processing method. The color difference map provided a unique response for each analyte. The useful information about chemical structure and intrinsic features of studied pesticides could be extracted from these patterns. The colorimetric profiles showed the additive details for the colorimetric detection. As clarified, the color of each sensing element became brighter from diazinon to malathion, i.e., the aliphatic pesticides had a strong interaction with NPs compared to heterocyclic compounds. Also, the tendency of the studied pesticides (malathion, parathion, and chlorpyrifos) for reaction with Tyr-modified AuNPs was higher than the other AuNPs.

The colorimetric procedure was repeated in two separate boxes with a length of 25 cm and 50 cm. The distance between spraying and sensing locations increased. As illustrated in Fig. S10, the color intensities of each sensing element diminished by increasing the length of the box. The total response of the sensor represented the assay ability to detect and discriminate the individual pesticides, even if the sensor was 50 cm far from the spreader.

### Discrimination analysis

**Figure 2:**
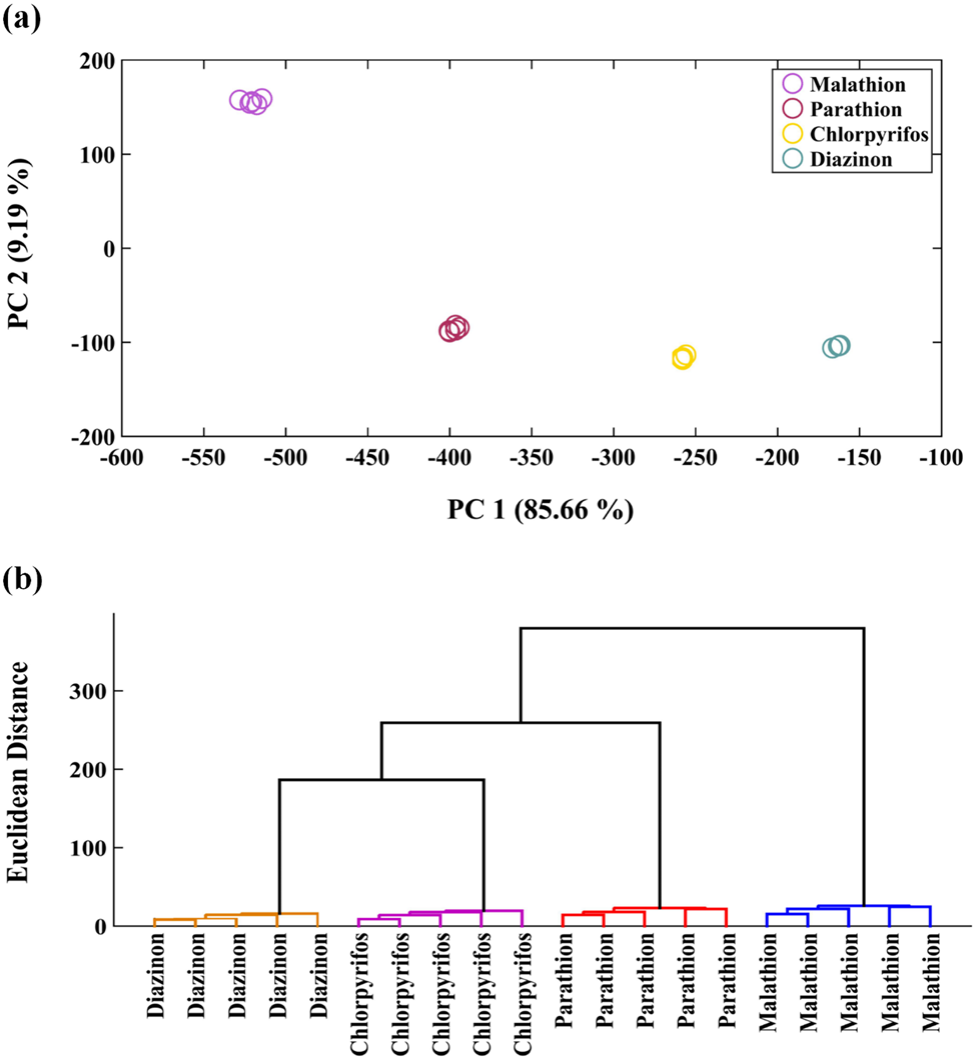
The discrimination of studied pesticides with two statistical pattern recognition methods: (**a**) principle component analysis (PCA) and (**b**) hierarchical clustering analysis (HCA). The experiment was performed in the Test box with different length (10 cm, 25 cm and 50 cm). The concentration of pesticides used in this study was 450.0 ng mL^−1^. Each sensing element was prepared by mixing 0.5 µL of borate buffer (0.1 M) with 0.4 µL of a certain NPs and 0.1 µL of deionized water. The pH of mixture was adjusted at 9.0.

As mentioned above, the designed PAD can provide unique analytical data for each studied pesticide so that it is possible to differentiate between the thion species. These differences are visible by the naked eye but must be confirmed by some pattern recognition methods. The color difference data were collected in a matrix with a size of 20 × 18 and entered principal component analysis (PCA) or hierarchical clustering analysis (HCA) as input data. The PCA score plot showed the extracted clusters embedded in a numerical data matrix, as in Fig. 2a. This Figure demonstrates that two first PCs included about 95% of total variances of raw data, 86% was distributed on PC 1 and the rest on PC 2. As observed, the thion clusters were well spaced apart and divided into families of aliphatic and aromatic. Further, the aromatic pesticides could be grouped into two individual classes, defined as heterocyclic (diazinon and chlorpyrifos) and non-heterocyclic (parathion). An excellent distinction was obtained between the heterocyclic analyte with a pyridine ring and that with a pyrimidine ring in its structure.

The statistical analysis was performed by a full dimensional method of HCA. The dendrograms provided by the HCA analysis (Fig. 2b) indicated the analytes categories in four clusters, belonging to aliphatic, aromatic, and heterocyclic pesticides.

These results were obtained when the concentration of each pesticide was equal to 450.0 ng mL^−1^. The qualitative studies of thions were repeated for the other concentrations, including 130.0 ng mL^−1^, 170.0 ng mL^−1^, 210 ng mL^−1^, and 250.0 ng mL^−1^. The dendrograms, as shown in Fig. S11, revealed that the analytes were individually separated at lower concentrations while the discrimination of families was achieved at values higher than 210.0 ng mL^−1^.

### Effect of interferences

To examine the selectivity of the assay, the PAD was subjected to other materials possibly present in the experiment media. The studied compounds were classified in the different categories, such as oxon organophosphates (paraoxon, dichlorvos, and trichlorfon), carbamate pesticides (carbaryl, pirimicarb, and carbofuran), alcohols (ethanol, methanol, and 1-hexanol), aldehyde (hexanal, heptanal, and benzaldehyde), amines (triethylamine, amylamine, benzylamine, pyridine, aniline, and ammonia), arenes (benzene, toluene, and *p*-xylene), acids (acetic acid, isobutyric acid, and phosphoric acid), alkanes (heptane, hexane, and heptane), and phosphine (dimethylphenylphosphine). The amount of these materials was similar to analytes concentration (450.0 ng mL^−1^). The colorimetric difference maps are indicated in Fig. S12. As seen, NPs did not aggregate when exposed to these interfering compounds, and no color change was observed. Besides, the studied thions with the concentration of 450.0 ng mL^−1^ were individually mixed with each foreign material at different concentrations. The assay responses to each mixture were determined and compared to that obtained with the only thion aerosols. Probably, The discrimination between thions from the other pesticide compounds is due to the presence of sulfur atoms in the thion structures. This atom has high affinity to not only metal core (gold and silver) but also the fictional groups in the chemical structure of capping agents. As provided in Table S3, all species did not interfere with this measurement; hence, the studied thions could be detected by the proposed assay with high selectivity.

### Determination analysis

**Figure 3:**
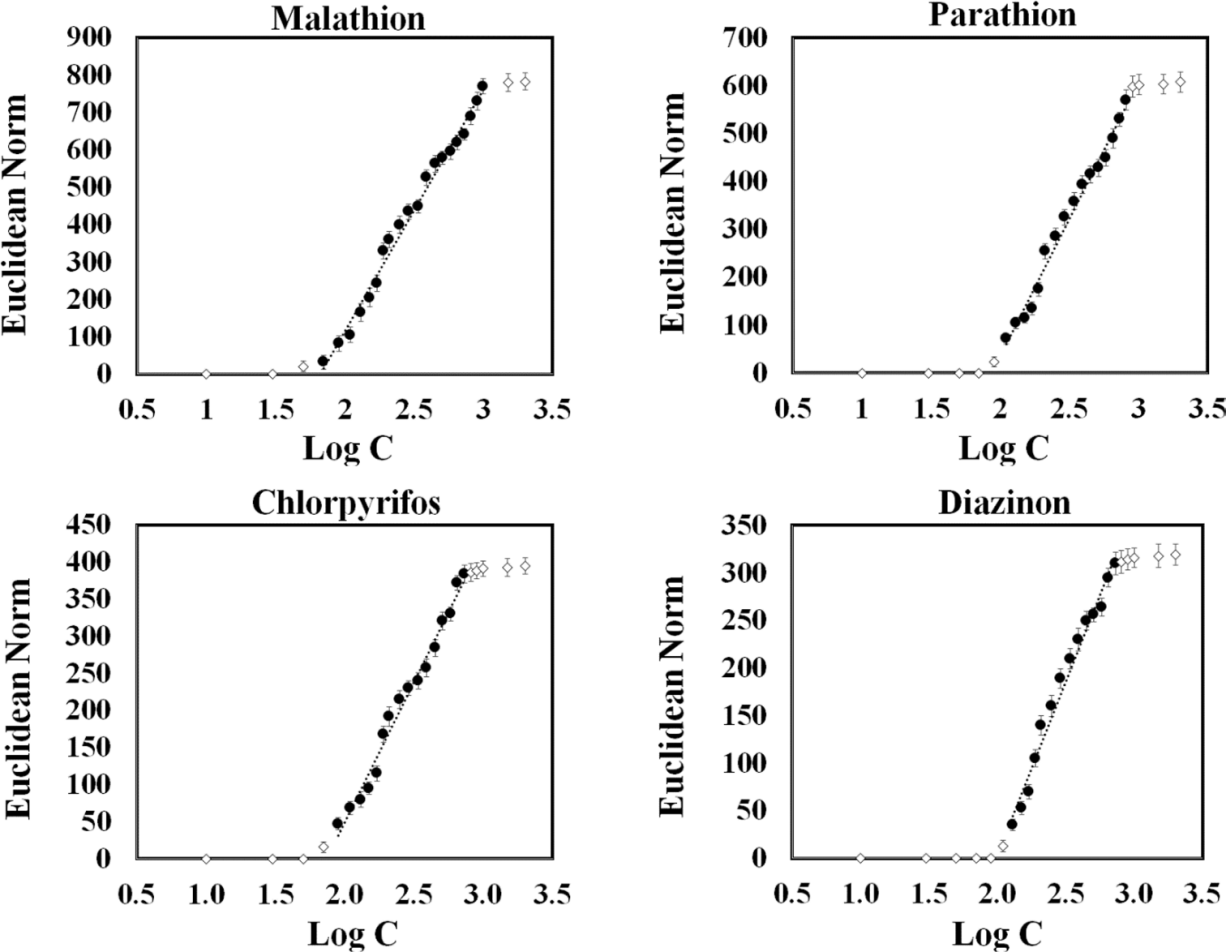
Correlation between the response of sensor (Euclidean norm) and the logarithm of concentrations for each studied pesticides. The linear range was shown with black solid circle. Each sensing element was prepared by mixing 0.5 µL of borate buffer (0.1 M) with 0.4 µL of a certain NPs and 0.1 µL of deionized water. The pH of mixture was adjusted at 9.0.

The efficiency of the proposed assay was examined on different amounts of pesticides with the analyte concentrations varied from 0 to 2.0 µg mL^−1^. Fig. S13 indicates the brightened color of each sensing element corresponding with the increment of analyte concentration. At specified concentration, a fingerprint colorimetric difference map was obtained for each thion. Therefore, the assay possible to distinguish the studied pesticides at the desired concentration. For these measurements, the calibration curves are given in Fig. 3 and additive analytical information can be found in Table 1. Obviously, the linear correlation was observed between the color changes and the pesticide values in the range of 70.0 ng mL^−1^–1000.0 ng mL^−1^ for malathion, 110.0 ng mL^−1^–810.0 ng mL^−1^ for parathion, 90.0 ng mL^−1^–730.0 ng mL^−1^ for chlorpyrifos, and 130.0 ng mL^−1^–730.0 ng mL^−1^ for diazinon. The limit of detection was calculated as 58.0, 103.0, 81.0 and 117.0 ng mL^−1^ for malathion, parathion, chlorpyrifos and diazinon, respectively.

**Table 1:** The analytical information for individual determination of studied pesticides.

Analyte	Linear range (ng mL^−1^)	Detection limit (ng mL^−1^)	R^2^
Malathion	70–1000.0	58.0	0.986
Parathion	110.0–810.0	103.0	0.984
Chlorpyrifos	90.0–730.0	81.0	0.983
Diazinon	130.0–730.0	117.0	0.980

**Figure 4:**
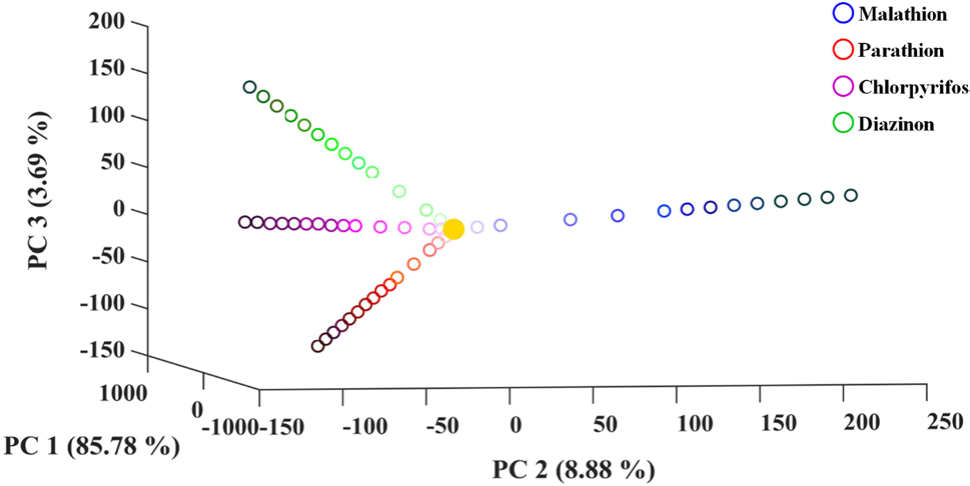
Discrimination analysis of studied pesticides with different concentrations. Principle component analysis (PCA) was used for this study. Each sensing element was prepared by mixing 0.5 µL of borate buffer (0.1 M) with 0.4 µL of a certain NPs and 0.1 µL of deionized water. The pH of mixture was adjusted at 9.0. The yellow solid circle is defined as the response of sensor before exposing to analyte.

The responses of all analytes were distributed in a space of three-dimension PCA score plot. The malathion at each concentration was completely discriminated from the other pesticides (Fig. 4). The aromatic compounds with a specified concentration were totally separated from each other. Also, a linear relationship was observed between the number of pesticides and values of principal components. It allows using the PCA score plot as a standard regression curve for estimating the concentration of analytes in the unknown samples.

### Reproducibility of assay responses

The reproducibility of the developed sensor was investigated by fabricating ten individual sensing array and exposing them to each pesticide with the concentration of 450.0 ng mL^−1^. Ten Euclidean norms of the response vector were determined for each analyte. The relative standard error (RSD) of these numerical data was calculated and listed in Table S4. The results show that amount of RSD % is lower than 10%. It means that the assay can detect a certain thion with acceptable reproducibility.

### Simultaneous analysis

**Figure 5:**
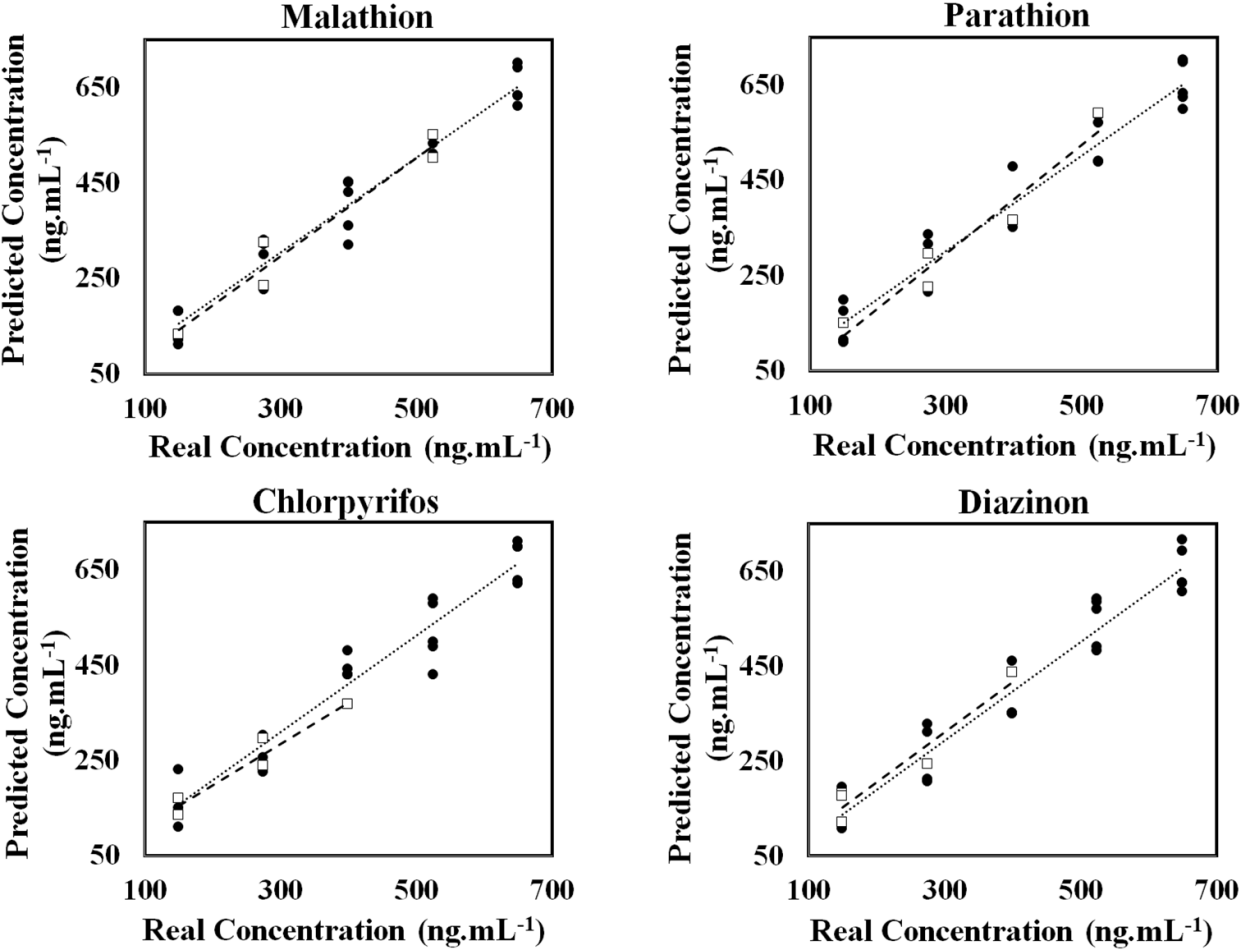
The results obtained by the PLS models for simultaneous analysis of the studied pesticides. In these graphs, training and prediction sets were shown with black solid circle and square markers, respectively. Each sensing element was prepared by mixing 0.5 µL of borate buffer (0.1 M) with 0.4 µL of a certain NPs and 0.1 µL of deionized water. The pH of mixture was adjusted at 9.0.

In practical, a real sample consists of different pesticides. Therefore, the developed assay need to test for simultaneous determination of diverse analytes. Thus, a set of standard mixtures was prepared using four pesticides with known concentrations. Then, a certain amount of analyte was selected from a credible guideline in each mixture (Table S5). The responses of assay corresponding to each mixture were monitored. The results were collected in a matrix with the size of 20 × 18 cm as a training set which was subjected to partial least square (PLS) as a multivariate statistical method to provide a regression model for each analyte. A validation set containing five mixing solutions of pesticide (Table S5) was used to check the reliability of the PLS model. Several optimal latent variables were detected by leave-one-out cross-validation (LOO-CV) method. The predicted pesticide concentrations obtained by the developed model were compared with corresponding real values by calculating some statistical methods such as root mean square errors (RMSE) and correlation coefficient (R^2^) (Table 2). Figure 5 illustrates the relationship between real and predicted concentrations of the analyte. The results show a good correlation coefficient and acceptable error rate for each analyte which prove a high potential to simultaneously analyze the thion pesticides in the mixture of the proposed assay.

**Table 2:** The analytical results for simultaneous analysis of four pesticides by using PLS multivariate regression models.

Analyte	PLS factor	RMSEC	RMSEP	R^2^_C_	R^2^_P_
Malathion	4	41.0	33.0	0.952	0.956
Parathion	4	46.0	42.0	0.939	0.942
Chlorpyrifos	4	49.0	45.0	0.930	0.936
Diazinon	4	51.0	43.0	0.923	0.925

Finally, the proposed PAD was compared with other E-noses which are fabricated by various sensing elements including enzyme-based sensor, fluorescent probe, commercial gas sensors, and chemical dyes. As illustrated in Table S6, only three works evaluated the discrimination of pesticides in the vapor phase. Among them, only the E-nose prepared by NPs was used for detection of thion pesticides. The fabricated sensor showed excellent accuracy for the classification of pesticides with both pattern recognition methods. Moreover, the NPs based E-nose represented a sensitive response to pesticide vapors. Unlike the previous reports, a filter paper was used as a substrate of the sensor, which was inexpensive and available.

## Conclusion

In this paper, an optical E-nose based on nanoparticles fabricating on a filter paper was developed successfully for pesticide aerosols analysis. The paper-based device was fabricated using 3D-origami pattern. In the detection spots, the NPs were formed using a low-cost chemical agent without the requirement of a biomaterial like an enzyme. The experiment results show clearly response for thion organophosphates, discriminated aliphatic, aromatic, and heterocyclic compounds from each other. The responses were not influenced by the other types of pesticides such as oxon organophosphate, carbamate, and different chemical organic compounds. The fabricated sensor showed acceptable performance for both individual and simultaneous quantitative analysis. This device can be embedded in the close, mask and skin of farmer as a diagnosis kit to monitor the pesticide pollution in agriculture environments.

## Method

### Materials

All compounds used in this study were in the analytical grades. The studied pesticides were malathion, parathion, paraoxon, dichlorvos, trichlorfon, carbaryl, pirimicarb, carbofuran chlorpyrifos, and diazinon. These materials and other chemicals, including cysteamine (Cys), tyrosine (Tyr), and tannic acid (TA), were purchased from Sigma Aldrich. Ethanol, methanol, 1-hexanol, hexanal, heptanal, benzaldehyde, triethylamine, amylamine, benzylamine, pyridine, aniline, ammonia, benzene, toluene, *p*-xylene, acetic acid, isobutyric acid, phosphoric acid, pentane, hexane, heptane, dimethylphenylphosphine, silver nitrate (AgNO_3_), gold (III) chloride trihydrate (HAuCl_4_·3H_2_O), sodium borohydride (NaBH_4_), boric acid, tris-hydroxymethyl methane (Tris), sodium hydroxide (NaOH), and hydrochloric acid (HCl) were obtained from Merck Chemical Company. Whatman Grade No. 2 filter paper was used as a sensor array substrate.

The standard solution of pesticide with a concentration of 30.0 µg mL^−1^ was made in ethanol. This solution was diluted by deionized water to prepare the pesticide solution with lower concentrations. The buffer was provided by dissolving a desirable amount of Tris or boric acid in a certain volume of deionized water. The pH of the buffer was adjusted at a specified value by adding drop by drop of NaOH and HCl solution (1.0 M).

### Instrument and software

UV/Vis spectrophotometer (P, Model V-570) was used to record the SPR spectra of synthesized nanoparticles. The modification of nanoparticles with a capping agent was evaluated by FT-IR spectroscopy [Thermo Scientific Nicolet IR100 (Madison, WI)]. The hydrodynamic size of nanoparticles and the electrical charge of their surface were determined by Zetasizer Nano ZS90 (Malvern, UK). The immobilization process of nanoparticles on the surface of the paper was investigated using field emission scanning electron microscopy (FE-SEM; MIRA3 TESCAN) and SEM-attached energy dispersed spectroscopy (EDX). The desired pattern of the sensor was designed in AutoCAD 2016 software (https://www.autodesk.com/products/autocad) and printed on the piece of paper by an HP LaserJet printer 1320. Changes in the color of nanoparticles were captured by a Canon EOS 750D digital camera. Image processing was performed by Image J software (1.51n, National Institutes of Health, USA) (https://imagej.nih.gov/ij/download.html). All discrimination and simultaneous quantitative analyses were done in the MATLAB R2015 scientific software (https://www.mathworks.com).

### Preparation of nanoparticles (NPs)

The sensor array was fabricated by six metallic NPs, including 3 gold NPs (AuNPs) and 3 silver NPs (AgNPs). The synthesized NPs were coated with 3 different stabilizing agents consisting of biogenic amine (cysteamine), amino acid (tyrosine), and polyphenol antioxidant (tannic acid). The procedures for the synthesis of NPs are described in the supplemental document (“Introduction”). The prepared NPs were characterized by spectrophotometric methods, and the results are represented in supporting information (“Results and discussion”).

### Design of paper-based Opto-E-nose

The desired pattern for the fabricated sensor array is shown in Fig. 1a. The device was made up of two zones, which are separated by a narrow line. Each zone had a hydrophilic site surrounded by hydrophobic barriers. The proposed pattern was depicted in the AutoCAD environment. The drawn pattern was projected on the Whatman filter paper by the ink-printing method. The paper was transferred into an oven with a temperature of 200 °C for 45 min^38^. During this time, the printer ink was melt and flowed in the layer of paper, filling the holes on that^38^. This process led to an increase in the hydrophobicity of barrier parts^38^. The sampling zone was a rectangular hydrophilic space. The detection zone included six circle-shaped hydrophilic sites. 1.0 µL aqueous solutions of NPs were injected into detection zones. The injection was performed by a digital micropipette. The tip of the micropipette was adjusted to the center of the zone. The solution was spread radially on the surface of the paper and took up the total space of zone. The elements of the sensor were provided by mixing the synthesized NPs with a buffer solution. The volume of NPs in the mixture, as well as type, pH, and concentration of buffer solution, was optimized. The image of the fabricated detection zone is shown in Fig. 1b.

### Sensing procedure

**Scheme 1:**
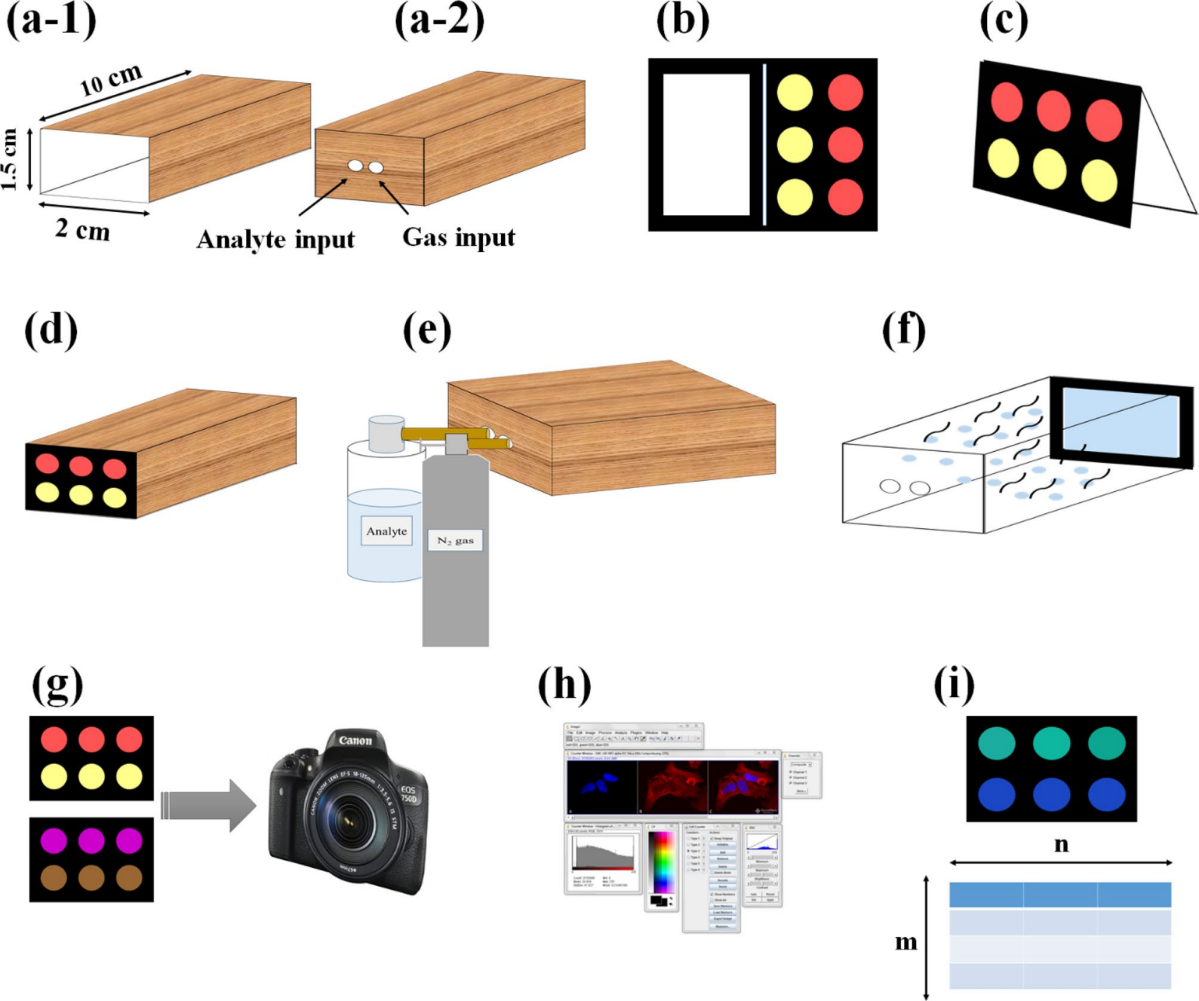
The schematic diagram for colorimetric procedure. (**a**) Fabricating a Test box with the size of (10 × 2 × 1.5), (**a-1**) the front face of Test box, (**a-2**) the behind face of Test box containing two holes to import analyte and carrier gas, (**b**) fabricating the paper based E-nose, (**c**) folding the PAD such that the sampling zone completely covered the detection zone, (**d**) embedding the PAD in the front face of Test box, (**e**) spraying the analyte and carrier gas to the Test box, (**f**) the stream of carrier gas including analyte aerosols which are directed towards sampling zone, (**g**) recording the image of PAD before and after exposing to analyte, (**h**) calculating RGB values of sensing elements by using image analysis software, (**i**) creating a color difference map and a data matrix sizing (m × n) for statistical analysis.

A general procedure for the colorimetric detection of pesticides is schematically shown in Scheme 1. The experiment was done in a text box, a cube with dimensions of 10 cm × 2 cm × 1.5 cm. The designing paper analytical device (PAD) was folded such that the sampling zone completely covered all the detection spots (Scheme 1b). The PAD was embedded in the square face of the box (Scheme 1c), and a hole was drilled on the opposite side (Scheme 1d). 5.0 mL of pesticide with a certain concentration was poured into a bottle connected to a hole by a plastic tube (Scheme 1e). The analyte was sprayed to the box and the pesticide aerosols released in the box space (Scheme 1f). The aerosols were directed toward the PAD using a stream of N_2_ gas (Scheme 1f). The pesticide aerosols were adsorbed by the sampling zone, immediately transferred to the detection zone, and simultaneously interacted with sensing elements (Scheme 1e). This procedure was repeatedly performed for five times. Note that, the flow of N_2_ gas was used as a control experiment in this study.

### Image processing and data analysis

For each experiment, the photo of the PAD was recorded by a digital camera before and after analysis. The captured photos were given to Image J software to calculate the mean of the color intensities of each sensing element. Three values corresponding to red, green, and blue color elements resulted for each color spot. Then, for each color element, the value of image before analysis was subtracted from that obtained by the image after analysis. Finally, 18 difference values (6 sensing elements × 3 color elements) were collected in a data vector. This process was conducted for both qualitative and quantitative analyses.

In the qualitative detection, the concentration of pesticides was equal to 450.0 ng mL^−1^. For each pesticide, five repeated measurements were performed, and the response vectors of the sensor were assembled in a data matrix with a size of 20 × 18. Two popular discrimination methods of principal component analysis (PCA) and hierarchical clustering analysis (HCA) were used to statistically evaluate the obtained matrix and find the unique clusters for each pesticide.

The quantitative analysis was done by employing pesticides at different concentrations in the range of 0–2.0 μg mL^−1^. For each concentration, a data vector was obtained. The Euclidean norm of this vector was determined as a total response of E-nose. The analytical features of the sensor were obtained by plotting the amount of Euclidean norm against the pesticide concentrations. The Euclidean norm is the total response of assay which is calculated from the following equation^37,39^:

$$ Euclidean\; Norm = \sqrt {{\mathop \sum \limits_{i}^{n} ((\Delta R_{i} )^{2} + (\Delta G_{i} )^{2} + (\Delta B_{i} )^{2} } )} $$

where, n is the number of sensing elements, i indicates the ith sensing elements in the array structure and ∆R, ∆G and ∆B are the difference color values of red, green and blue elements for each sensing elements, respectively.

Finally, the assay was applied to determine simultaneously four thion pesticides in a mixture. The analysis was performed by the principle of partial least square (PLS) method. The PLS model was created by a training set containing 20 standard mixture of four pesticides. The accuracy of the developed model was investigated by five individual mixtures collected in a validation set. The concentration of each pesticide in a certain mixture was set by multilevel partial factorial design reported by Brereton^36^.

## Supplementary information

Supplementary Information.
