# Supplementary material for: Optoelectronic nose based on an origami paper sensor for selective detection of pesticide aerosols
Source: Sci Rep. 2020 Oct 14;10:17302. doi: 10.1038/s41598-020-74509-8 (PMC7560735; doi:10.1038/s41598-020-74509-8)
Supplement: Supplementary file 1 — Supplementary Information. [file 41598_2020_74509_MOESM1_ESM.docx]

**Supporting information**

**for**

**Optoelectronic nose based on an origami paper sensor for selective detection of pesticide aerosols**

Mohammad Mahdi Bordbar^1^, Tien-Anh Nguyen^2^, Anh Quang Tran^3^, Hasan Bagheri ^1,*^

*^1)^ Chemical Injuries Research Center, Systems Biology and Poising Institute, Baqiyatallah University of Medical Sciences, Tehran, Iran.*

*^2)^ Department of Physics, Le Quy Don Technical University, Ha Noi, Viet Nam.*

*^3)^ Department of Biomedical Engineering, Le Quy Don Technical University, Ha Noi, Viet Nam.*

**1. Procedures for the synthesis of NPs**

The studied NPs were prepared through the standard methods fully explained in the following:

**Cys-AuNPs.** An aqueous solution of HAuCl_4_ (40.0 mL, 1.4 × 10^-3^ M) was mixed with 400.0 µL of cysteamine aqueous solution with a concentration of 2.13 × 10^-1^ M. The mixture was kept on the stirrer at 25°C for 25 min. Then, 10.0 µL of the aqueous solution of NaBH_4_ (1.0 × 10^-2^ M) was added to the previous mixture drop by drop. The resulting solution was stored in the dark condition on the stirrer with vigorous rotation. The experiment continued at room temperature for 25 min. In this period, the solution color changed from yellow to red, which is a reason for the formation of NPs ^1^.

**Tyr-AuNPs.** An aqueous solution of HAuCl_4_ with a concentration of 1.0 × 10^-4^ M was added dropwise to 100.0 mL mixed solution of tyrosine and potassium hydroxide with a concentration of 1.0 × 10^-4^ M and 1.0 × 10^-3^ M, respectively. The alkaline solution of tyrosine was boiled previously. The experiment was done under a stirring condition. After the synthesis of NPs, the colloidal solution was dialyzed by 12 kDa dialysis membrane to remove unreacted metal ions and tyrosine ^2^.

**TA-AuNPs.** 50.0 mL of HAuCl_4_ aqueous solution (1.3 × 10^-4^ M) was heated to boil; the pH of the HAuCl_4_ solution should be adjusted at 6.0 before heating. This boiling solution was mixed with 2.0 mL of an aqueous solution of tannic acid with a concentration of (6.0 × 10^-3^ M). The mixture was kept under the previous condition for 2 min. Then, the solution was stirred at room temperature for 10 min ^3^.

**Cys-AgNPs.** First, a mixture containing cysteamine (6.0 mL, 0.3 % W/V) and AgNO_3_ (40.0 mL, 1.0 × 10^-4^ M) was prepared and vigorously stirred in an ice bath for 20 min. Next, the ice-cold NaBH_4_ solution (30.0 mL 4.0 × 10^-4^ M) was added drop by drop to the mixed solution. The experiment was performed under a stirring condition. Then, the mixture was allowed to reach room temperature and stored on the stirrer for 60 min. During this time, the solution turned to yellow ^4^.

**Tyr-AgNPs.** The procedure started by boiling 100.0 mL of an aqueous solution containing tyrosine (1.0 × 10^-4^ M) and potassium hydroxide (1.0 × 10^-3^ M). Then, the aqueous solution of AgNO_3_ (1.0 × 10^-4^ M) was mixed drop by drop with the boiling solution. This work continued until the solution turned to yellow. In order to provide a clean NPs solution and eliminate the unbounded materials, the dialysis membrane was used to dialyze the resulting solution ^5^.

**TA-AgNPs.** To perform this synthesis, the solutions of tannic acid (6.0 × 10^-2^ M) and AgNO_3_ (3.0 × 10^-3^ M) were prepared separately. The pH of the tannic acid solution was adjusted at 8.0 for further study. 2.0 mL of tannic acid solution (pH 8.0) was mixed with 5.0 mL of the AgNO_3_ solution, and the mixture was stored under the stirring condition at room temperature. Under this condition, the color of the solution gradually turned to yellow. The prepared solution was kept on the stirrer for 12 h ^6^.

**2. Characterization of synthesized NPs**

The main features of prepared NPs were verified by spectrophotometric methods. The absorption spectra obtained by the UV-Vis spectrophotometer are shown in Fig. S14. As seen, the SPR peaks for AgNPs were located in the wavelength range of 400 nm to 500 nm, while the maximum bonds of AuNPs appeared in the range of 500 nm to 600 nm. This figure shows that AgNPs stabilized by Cys, Tyr, and TA had a maximum absorbance at 405 nm, 425 nm, and 410 nm, respectively. Also, the specified peaks placed at 525 nm, 520 nm, and 535 nm were assigned to AuNPs modified by Cys, Tyr, and TA.

To evaluate the modification surface of NPs by capping agents, the FT-IR spectra of chemical compounds were compared with those obtained by synthesized NPs. As illustrated in Fig. S15, the certain absorption bands of stabilizing agent appeared in the spectrum of respective NPs. However, the intensity of the absorption peaks in the nanoparticle spectrum decreased, or its position shifted to higher wavelengths. This confirmed the capping agent participation in the formation of NPs structure. In the following, the FT-IR spectrum of each material was described in detail. In Fig. S15, the particular bands of Cys were observed at 3000 cm^-1^ to 3300 cm^-1^ for N-H stretching vibration, 2340 cm^-1^ to 2360 cm^-1^ for S-H stretching vibration, 1580 cm^-1^ to 1650 cm^-1^ for N-H bending vibration, and 1250 cm^-1^ to 1330 cm^-1^ for C-H bending vibration ^7^. The intense absorption peaks in the Tyr spectrum were 3400 cm^-1^, 3100 cm^-1^, 2800 cm^-1^, and 1650 cm^-1^ for O-H, N-H, and C-H stretching vibrations. Also, the bands located at 1584 cm^-1^ and 1404 cm^-1^ belonged to N-H bending and C-C aromatic starching vibrations, respectively ^8^. The spectrum of TA included sharp IR bands at 3400 cm^-1^ for O-H stretching vibrations. The other peaks were located at 1700 cm^-1^, 1610 cm^-1^, and 1330 cm^-1^ for C=O, C=C, and C-O stretching vibrations. The bending vibrations of C-O-H, C-O-C, O-H, and C-H caused the specified peaks at 1440 cm^-1^, 1026 cm^-1^, 875 cm^-1^, and 758 cm^-1^ ^3^.

The average size of synthesized NPs was estimated by dynamic light scattering (DLS). Fig. S16 shows that the hydrodynamic size of NPs increased from 26 nm for Cys-AgNPs to 48 nm for TA-AuNPs. The size distribution of the other NPs was placed in this range. As clarified, the average size of Cys-AuNPs, Tyr-AgNPs, Tyr-AuNPs, and TA-AgNPs was equal to 36 nm, 30 nm, 36 nm, and 42 nm, respectively.

The distribution of electrical charges on the surface of NPs was investigated by determining Zeta potential. The graphs in Fig. S17 indicate that four synthesized NPs, including Cys-AgNPs, Cys-AuNPs, TA-AgNPs, and TA-AuNPs, had an electrically positive surface. The zeta potential of these NPs was calculated as +42 mV, +38 mV, +13 mV, and +18 mV, respectively. On the other hand, the surface of both NPs modified by tyrosine was extremely negative. For these NPs, the respective zeta potential was -44 mV and -42 mV.


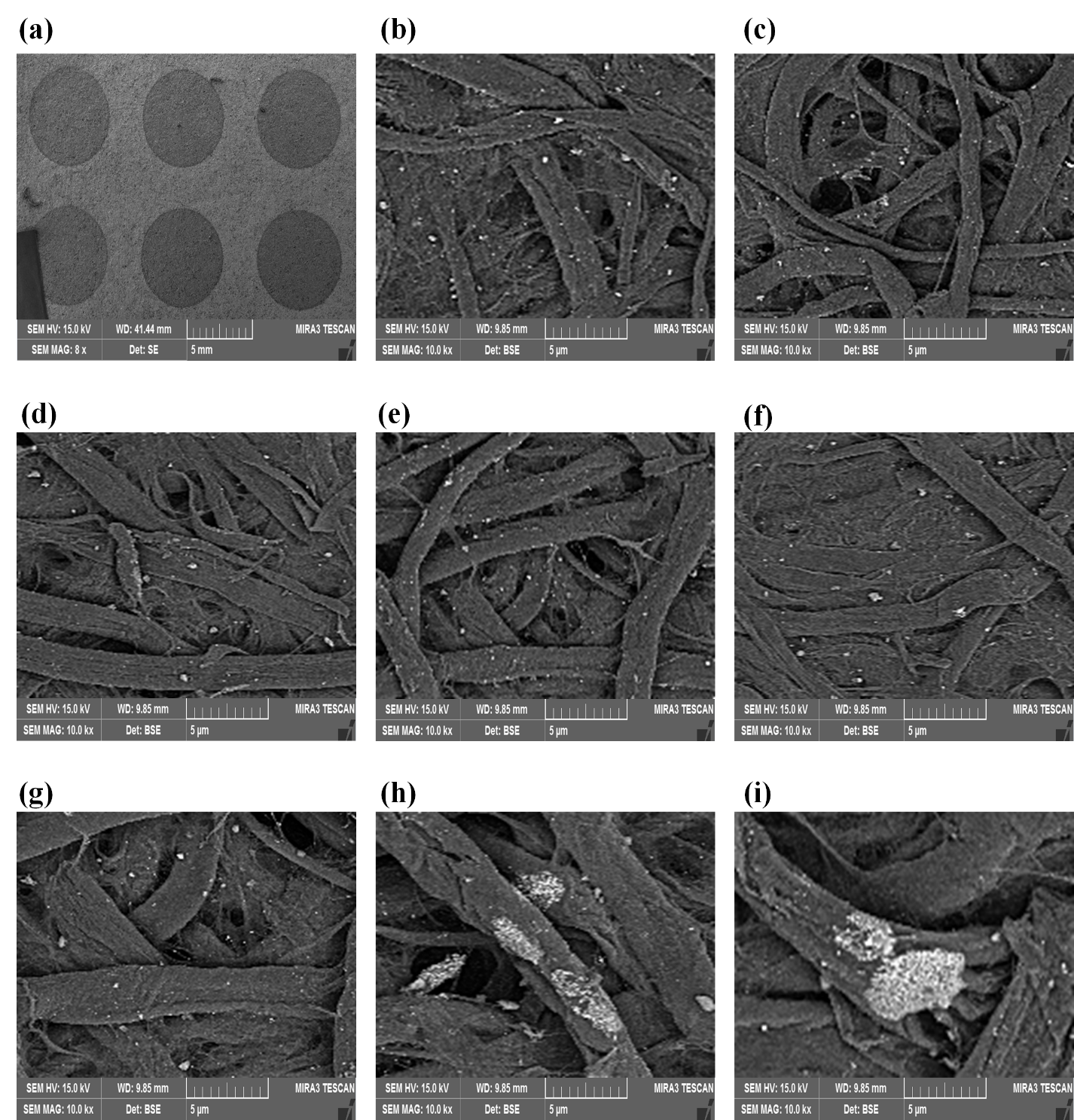


**Figure S1.** The SEM images of paper based E-nose. (a) The whole image of the sensor including the six spot of sensing elements, (b-g) distribution of NPs on corresponding hydrophilic zones. The aggregation of NPs in the presence of malathion was shown in (h) and (i) for Cys-AuNPs and Cys-AgNPs, respectively.


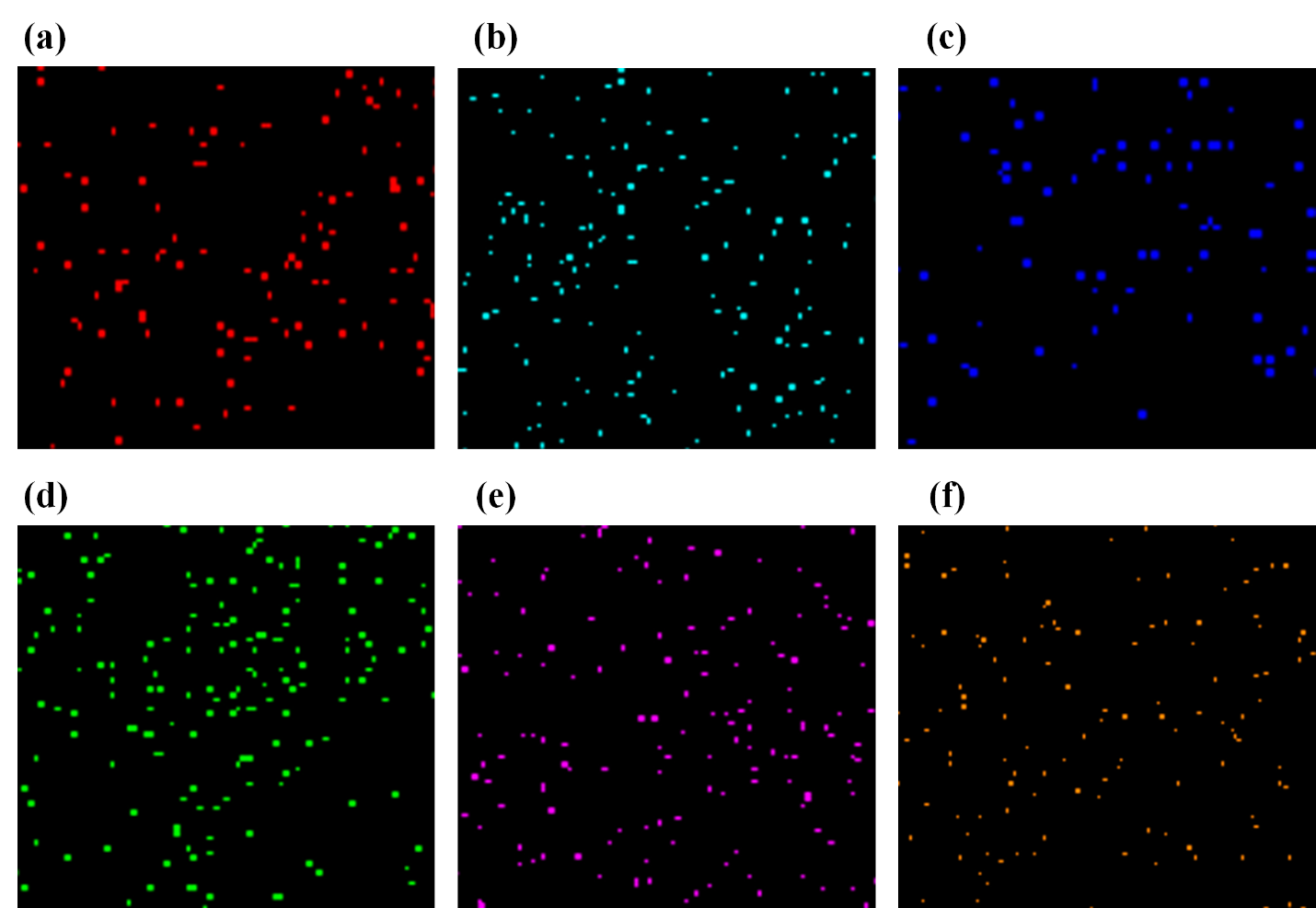


**Figure S2.** The SEM mapping images of detection zones. The distribution of NPs on the corresponding hydrophilic zones. (a) Cys-AuNPs, (b) Tyr-AuNPs, (c) TA-AuNPs, (d) Cys-AgNPs, (e) Tyr-AgNPs and (f) TA-AgNPs.


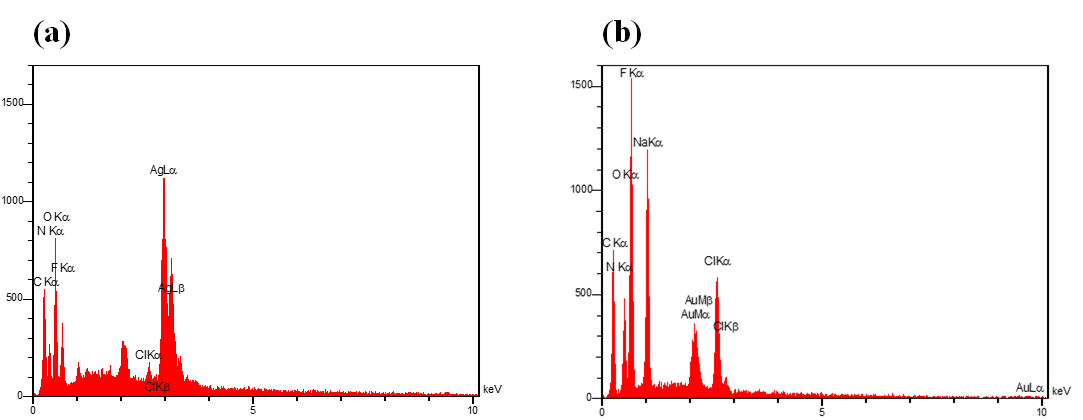


**Figure S3.** The EDX spectra of AgNPs and AuNPs prepared by Cys,


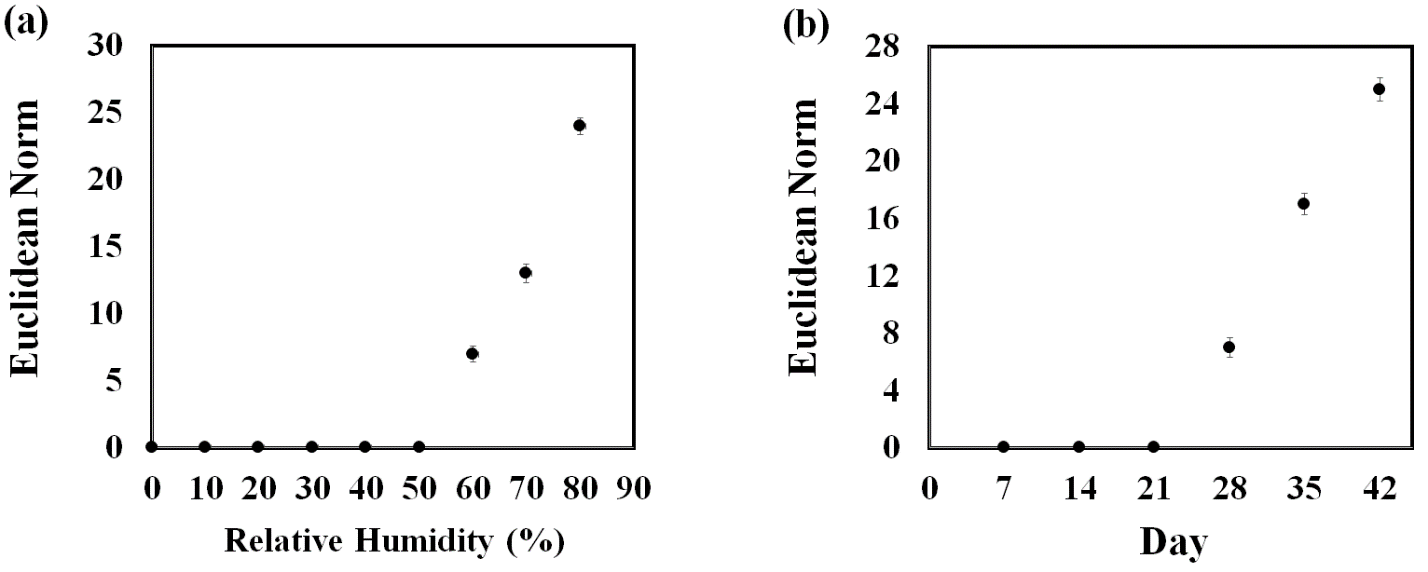


**Figure S4.** (a) Changes in the color of sensor array in different ambient humidity, (b) Evaluating the stability of sensing element before using for analysis.


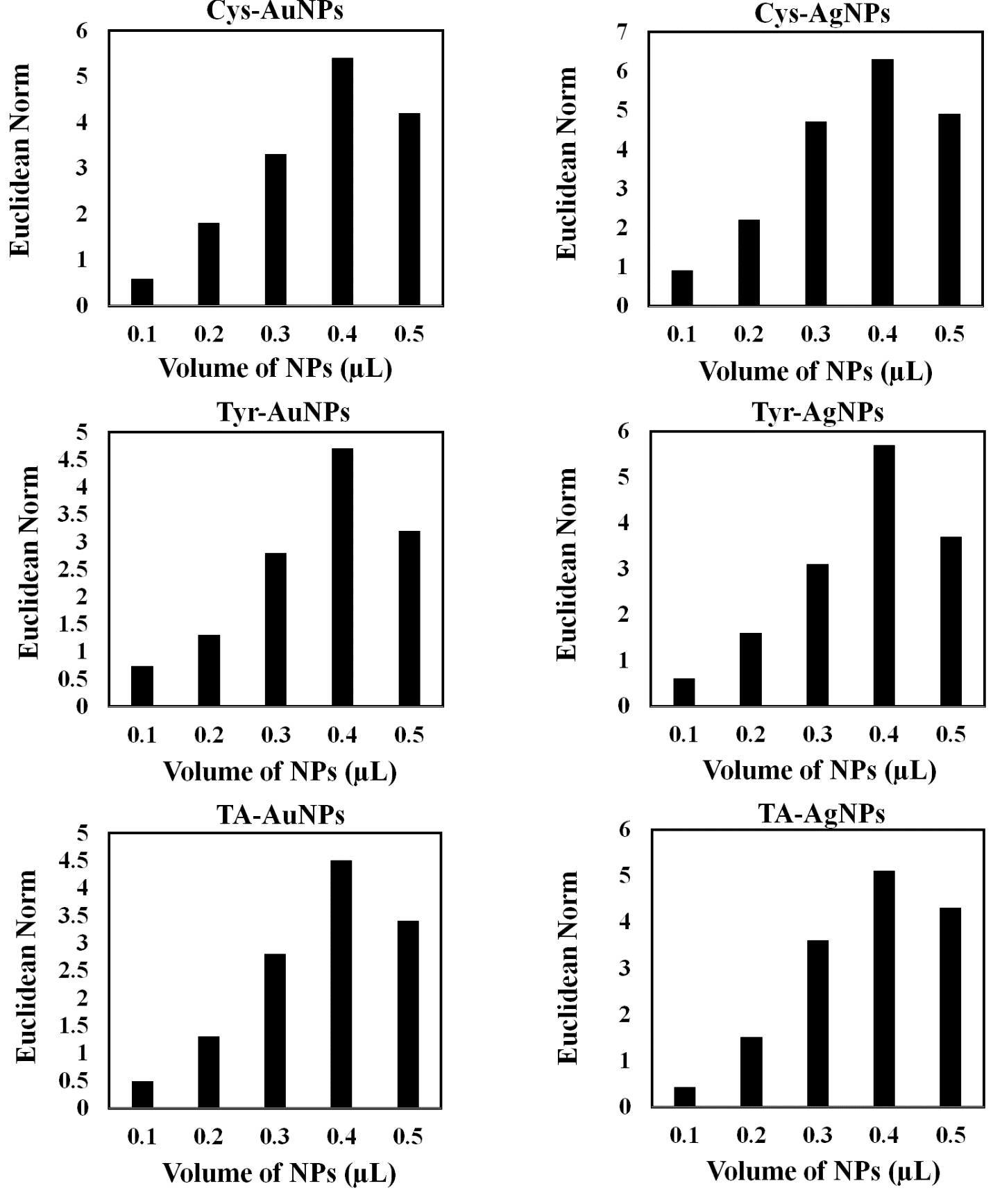


**Figure S5.** Finding the optimum volume of NPs by using discrimination ability function (DAF). The concentration of pesticides used in this study was 450.0 ng.mL^-1^.


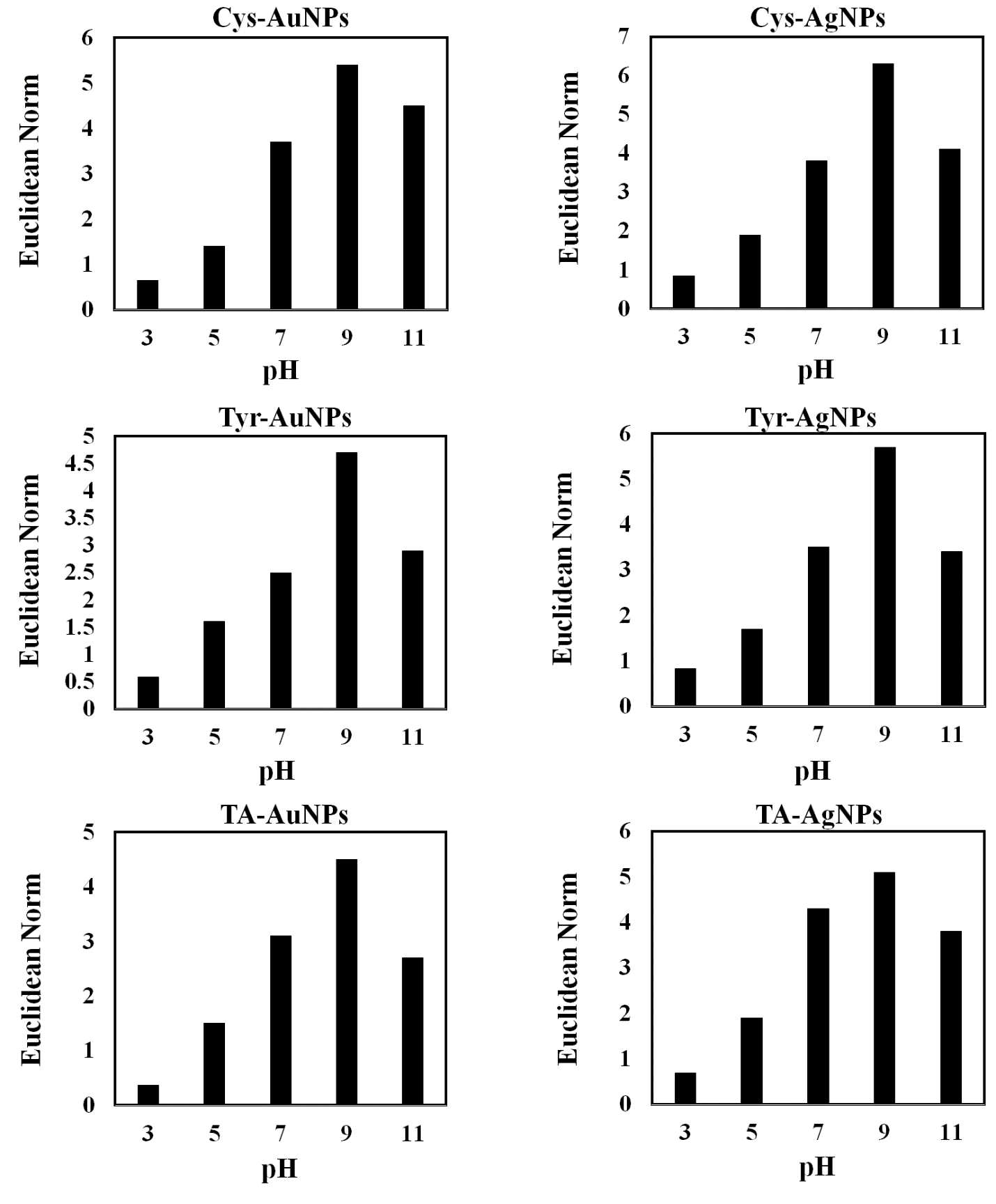


**Figure S6.** Finding the optimum pH of media by using discrimination ability function (DAF). The concentration of pesticides used in this study was 450.0 ng.mL^-1^ and the volume of NPs was equal to 0.4 µL for each sensing elements.


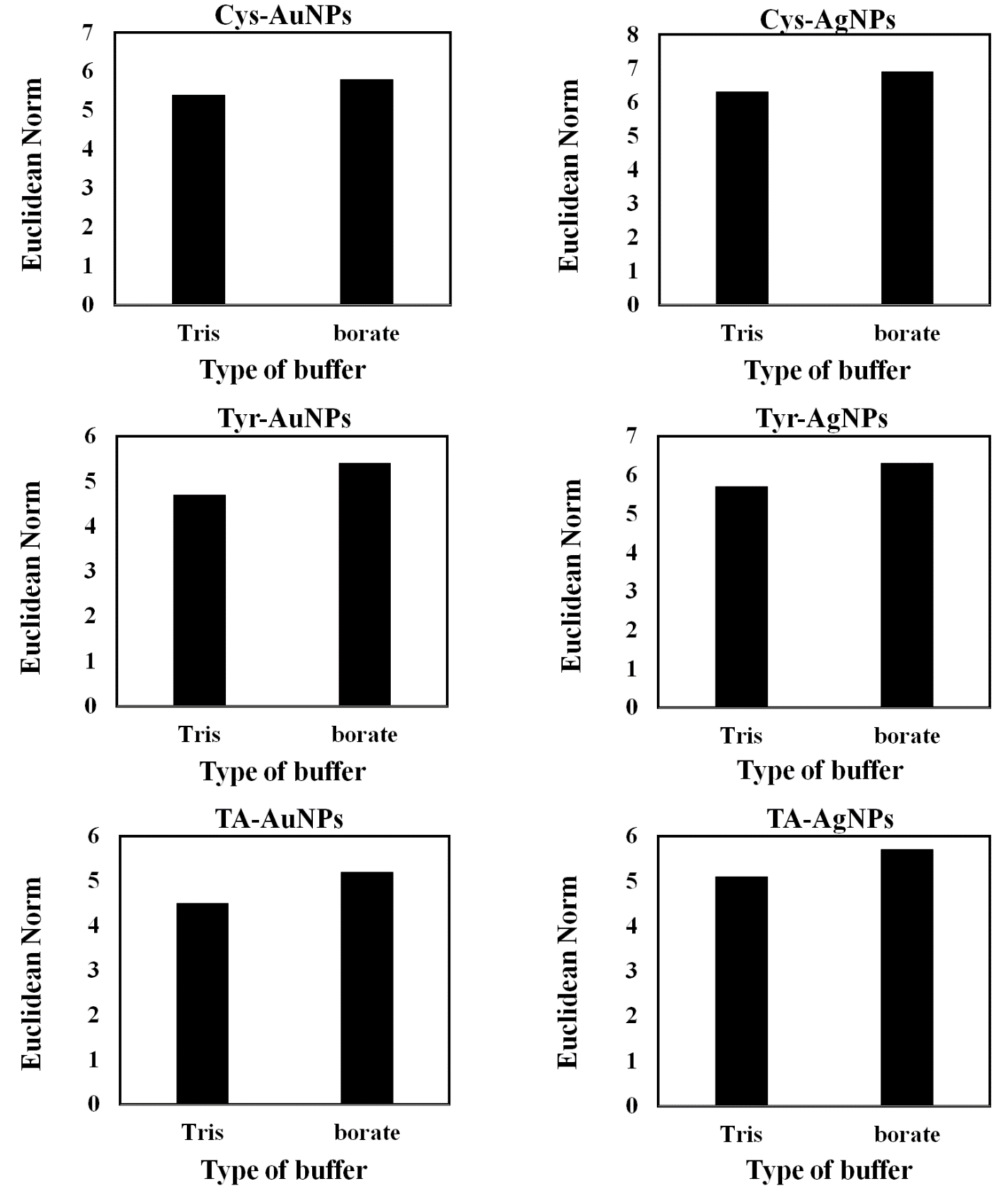


**Figure S7.** Finding the optimum type of buffer by using discrimination ability function (DAF). The concentration of pesticides used in this study was 450.0 ng.mL^-1^, the volume of NPs was equal to 0.4 µL for each sensing elements and the pH of buffer was adjusted at 9.0.


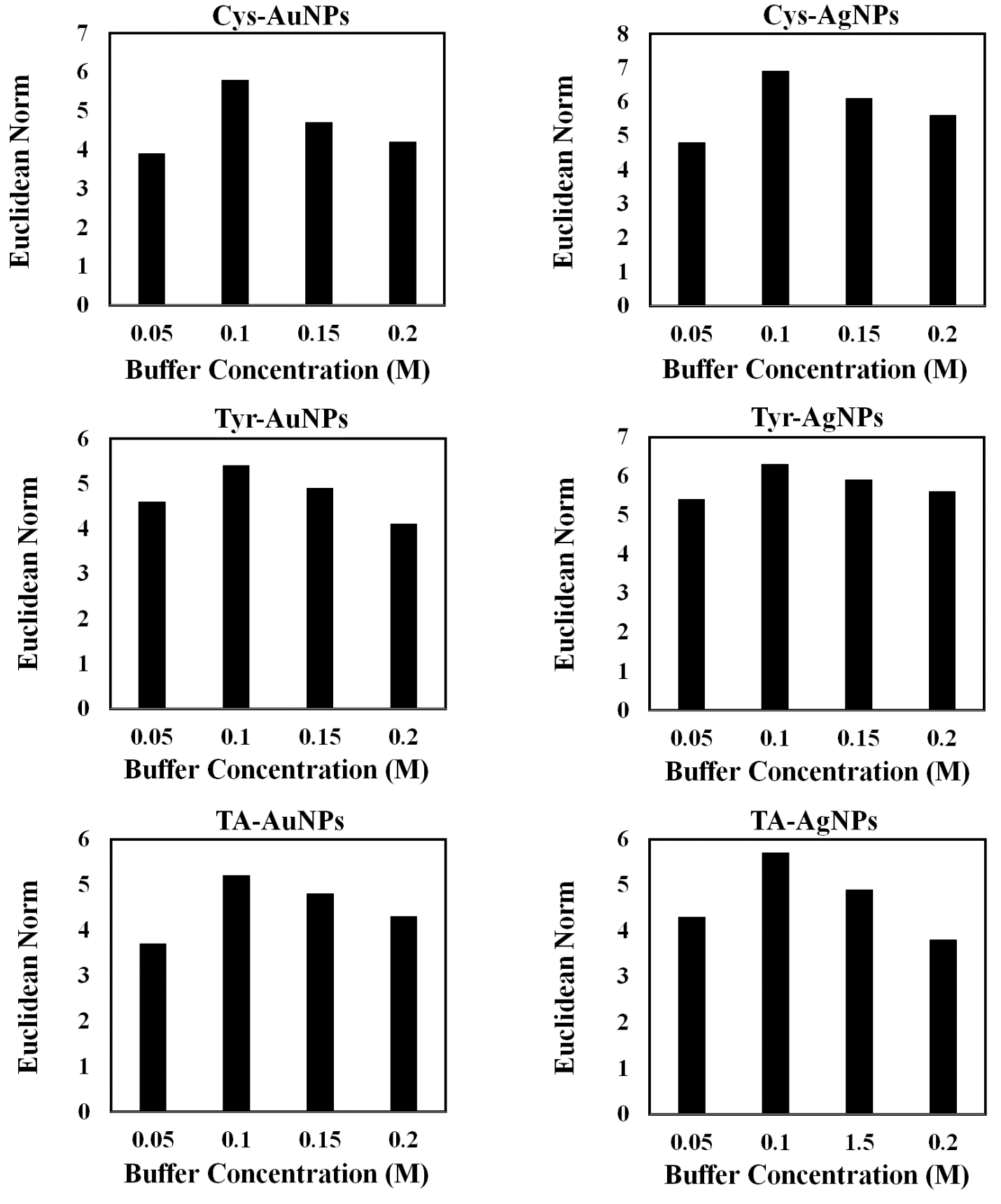


**Figure S8.** Finding the optimum concentration of buffer by using discrimination ability function (DAF). The concentration of pesticides used in this study was 450.0 ng.mL^-1^, the volume of NPs was equal to 0.4 µL for each sensing elements and the pH of borate buffer was adjusted at 9.0.


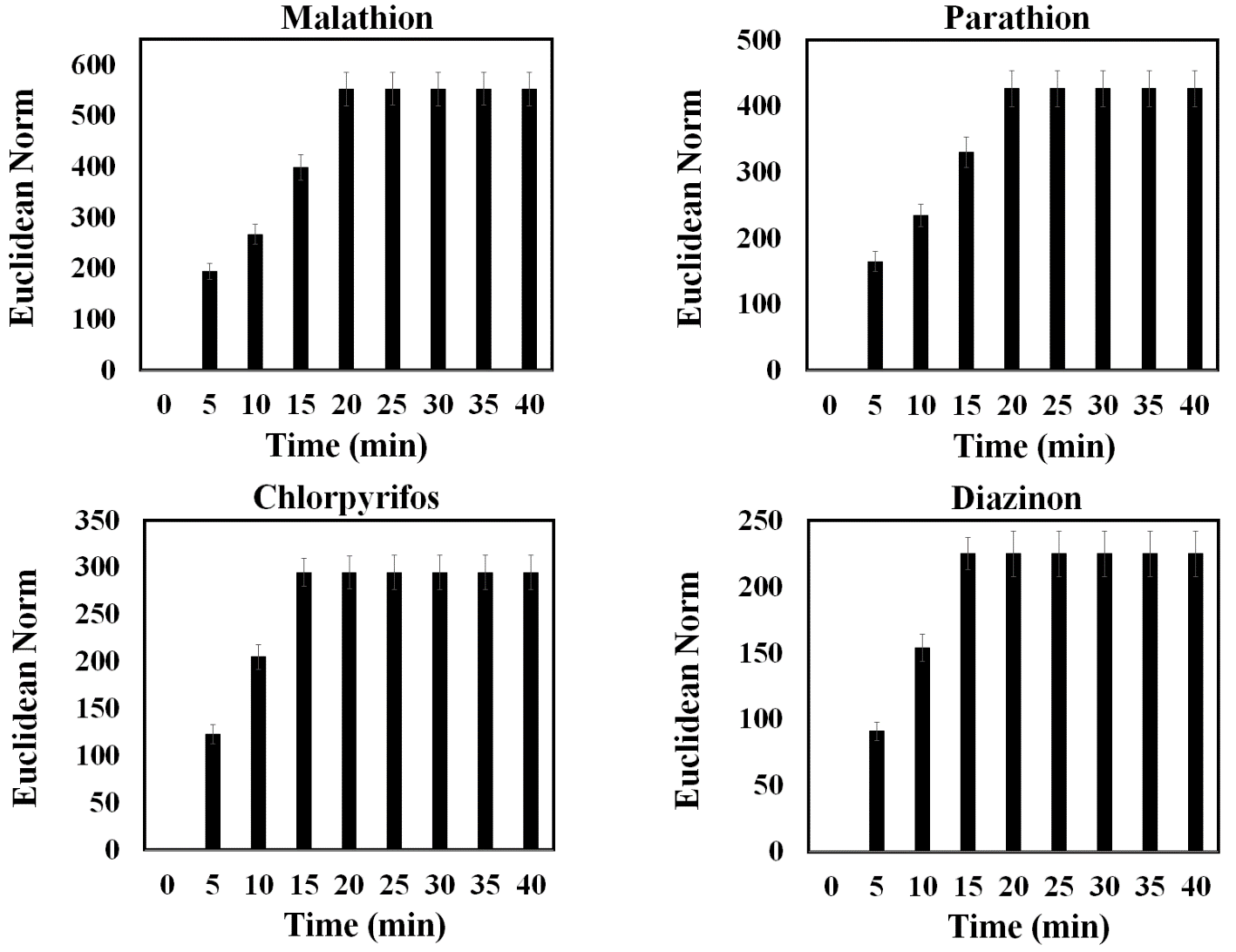


**Figure S9.** Finding the optimum incubation time for interaction of NPs and each studied pesticides. The concentration of pesticides used in this study was 450.0 ng.mL^-1^. Each sensing element was prepared by mixing 0.5 µL of borate buffer (0.1 M) with 0.4 µL of a certain NPs and 0.1 µL of deionized water. The pH of mixture was adjusted at 9.0.


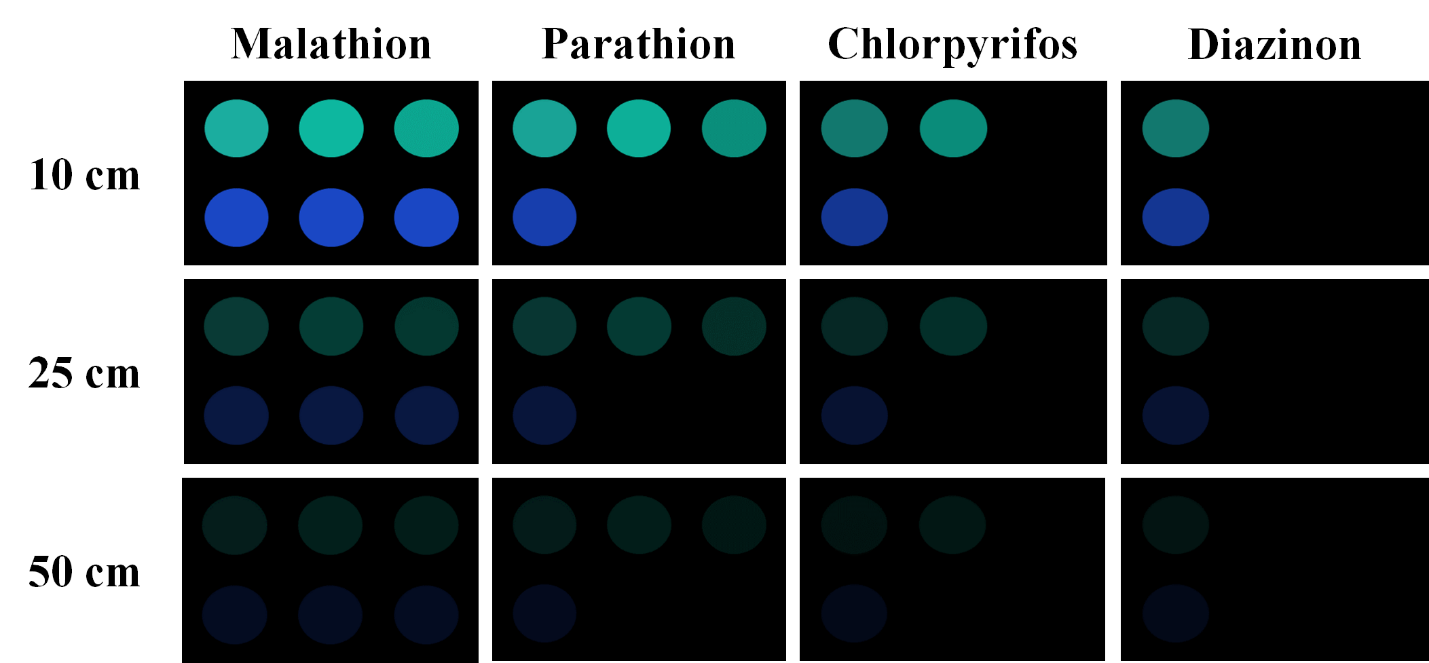


**Figure S10.** The response of sensor in the presence of studied pesticides. The experiment was performed in the Test box with different length (10 cm, 25 cm and 50 cm). The concentration of pesticides used in this study was 450.0 ng.mL^-1^. Each sensing element was prepared by mixing 0.5 µL of borate buffer (0.1 M) with 0.4 µL of a certain NPs and 0.1 µL of deionized water. The pH of mixture was adjusted at 9.0.


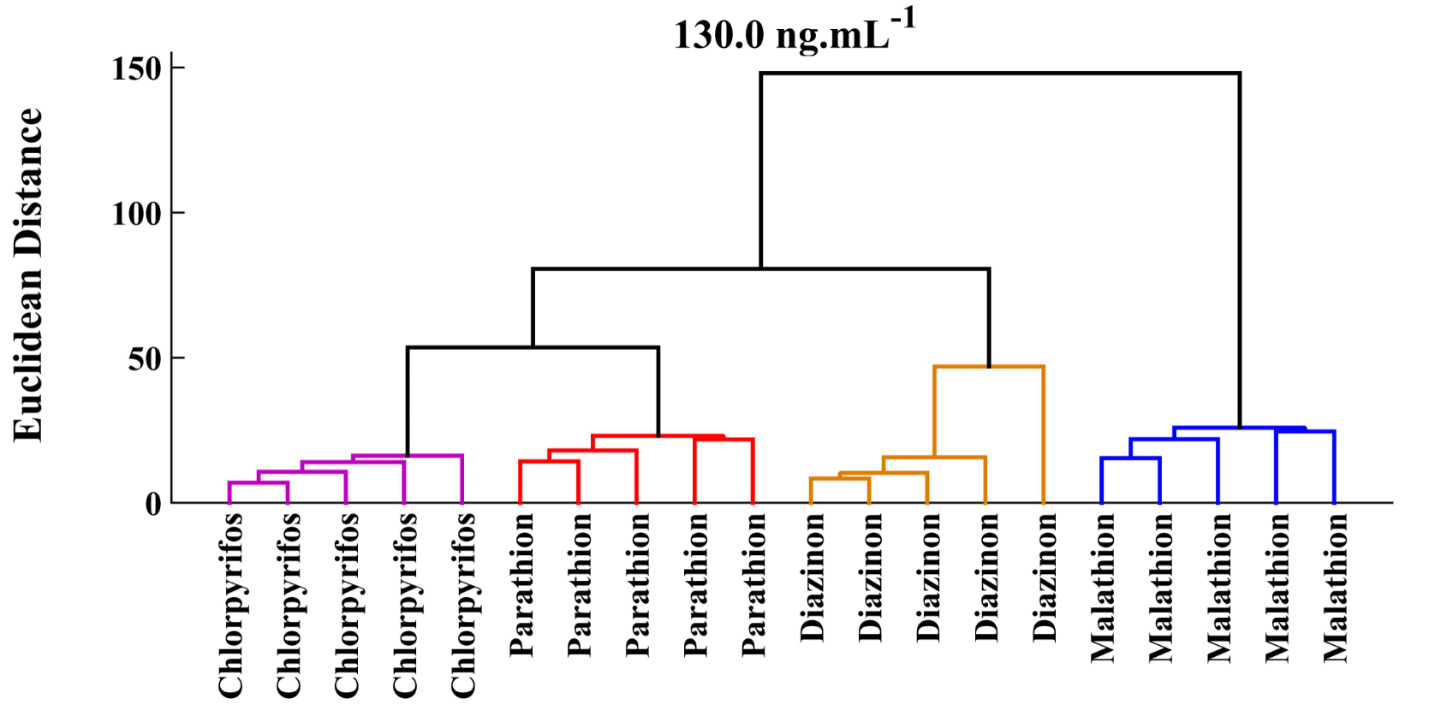


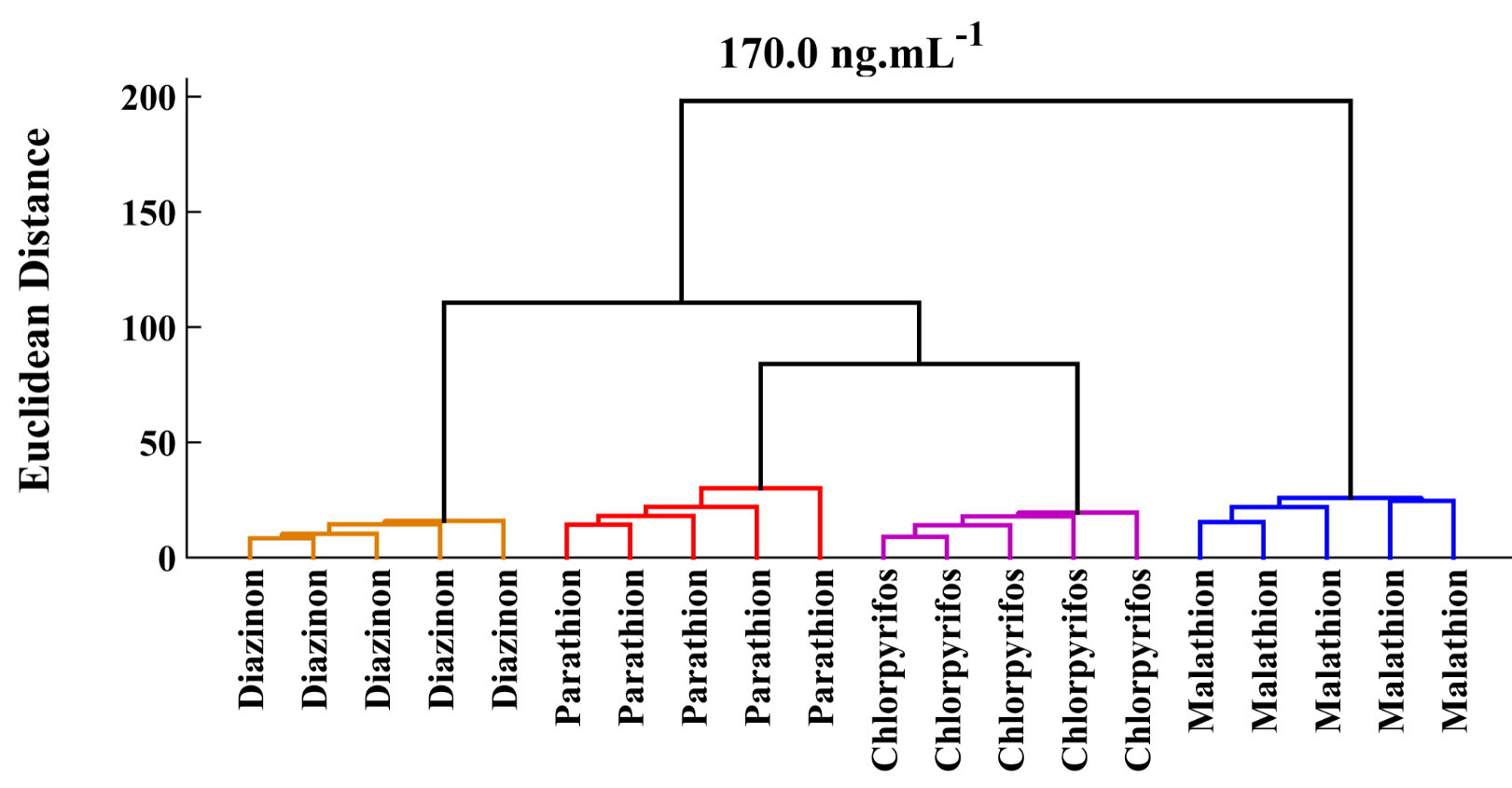


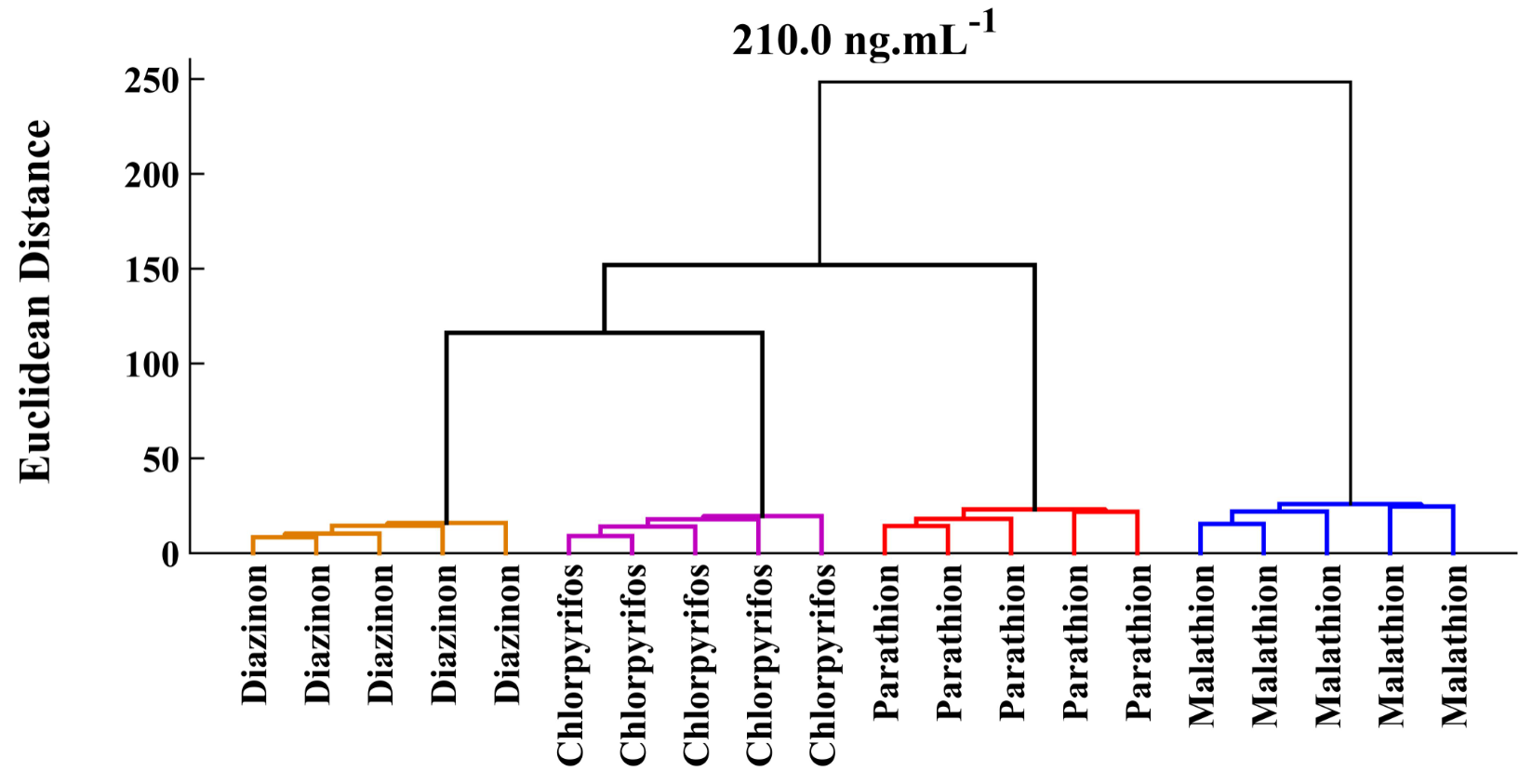


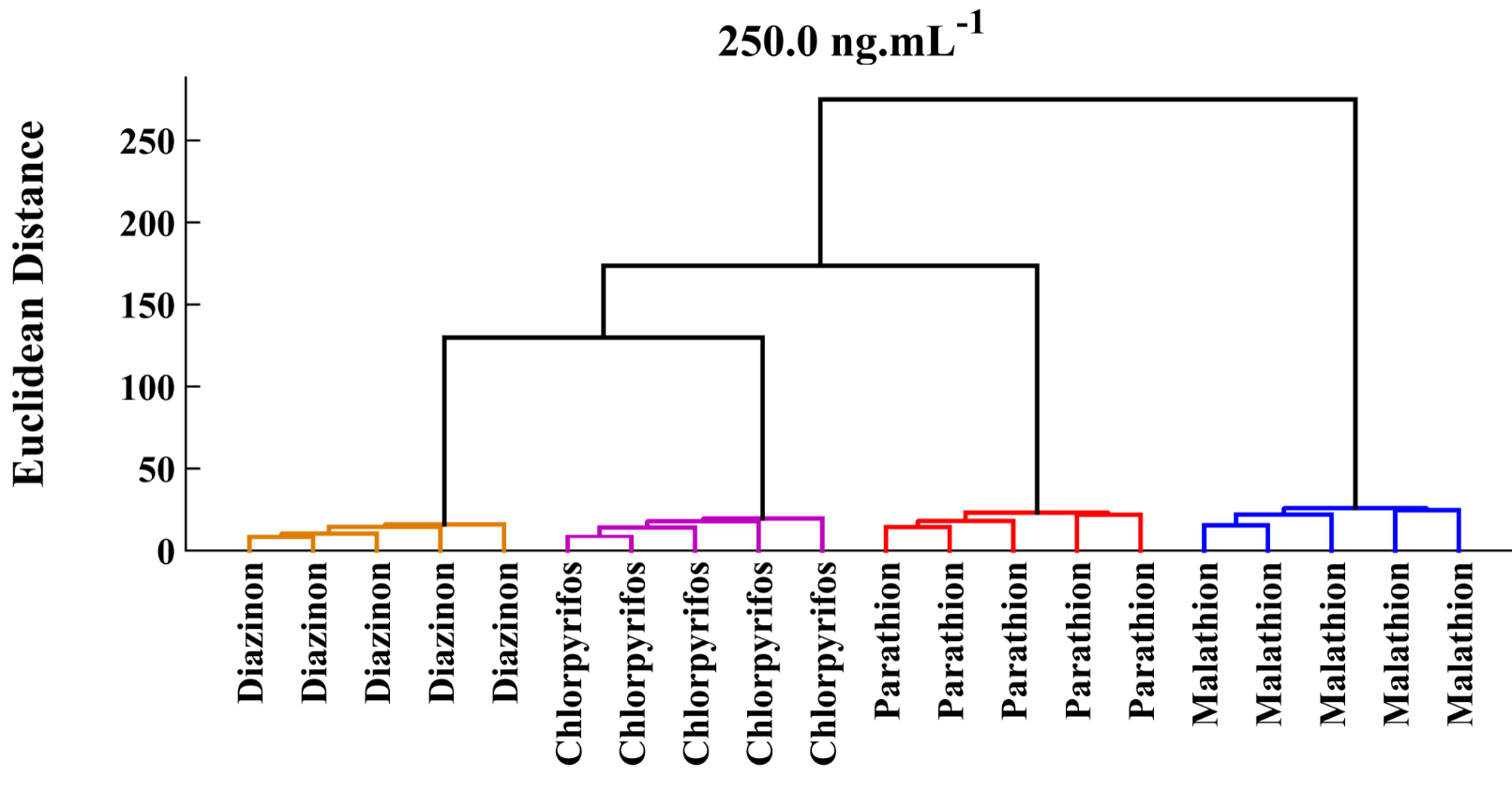


**Figure S11.** Evaluation of the discriminatory ability of sensor array at different concentration of studied pesticides. Hierarchical clustering analysis (HCA) was used for this study. Each sensing element was prepared by mixing 0.5 µL of borate buffer (0.1 M) with 0.4 µL of a certain NPs and 0.1 µL of deionized water. The pH of mixture was adjusted at 9.0.


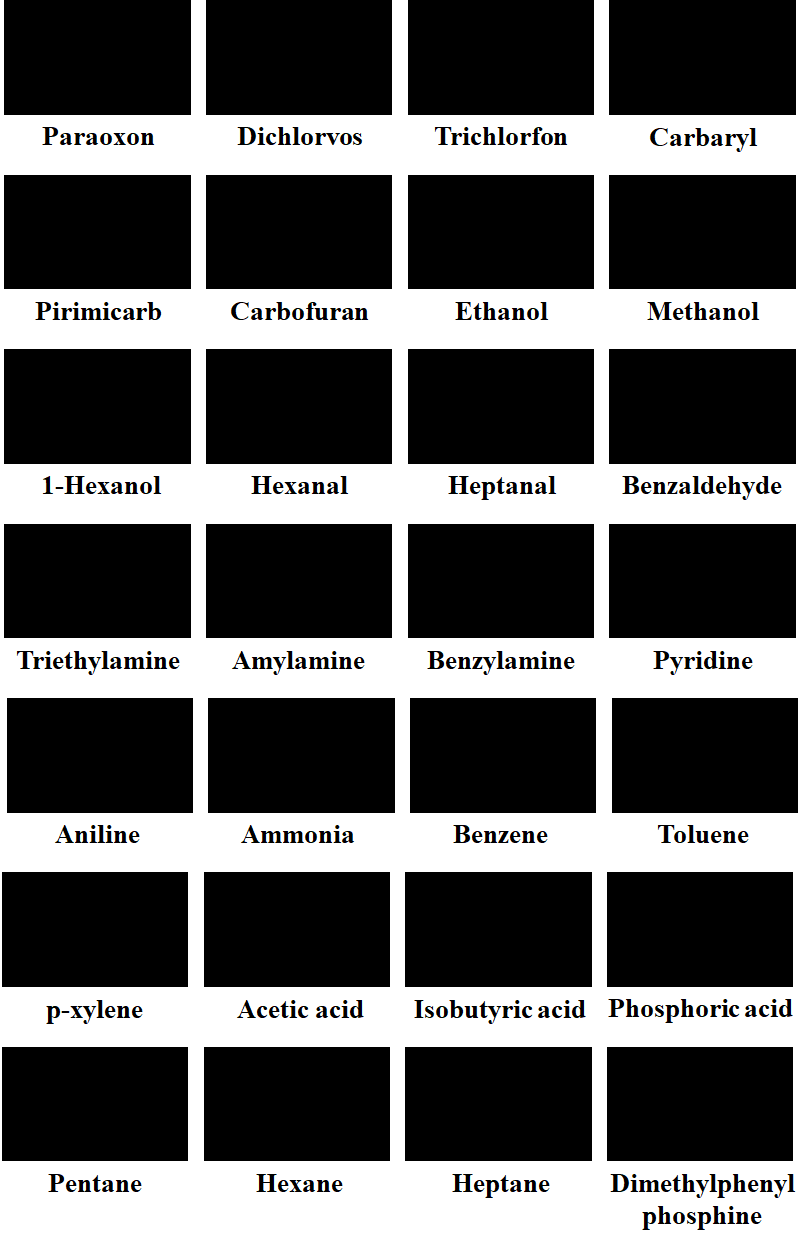


**Figure S12.** The colorimetric difference maps for interfering materials with the concentration of 450.0 ng.mL^-1^. Each sensing element was prepared by mixing 0.5 µL of borate buffer (0.1 M) with 0.4 µL of a certain NPs and 0.1 µL of deionized water. The pH of mixture was adjusted at 9.0.


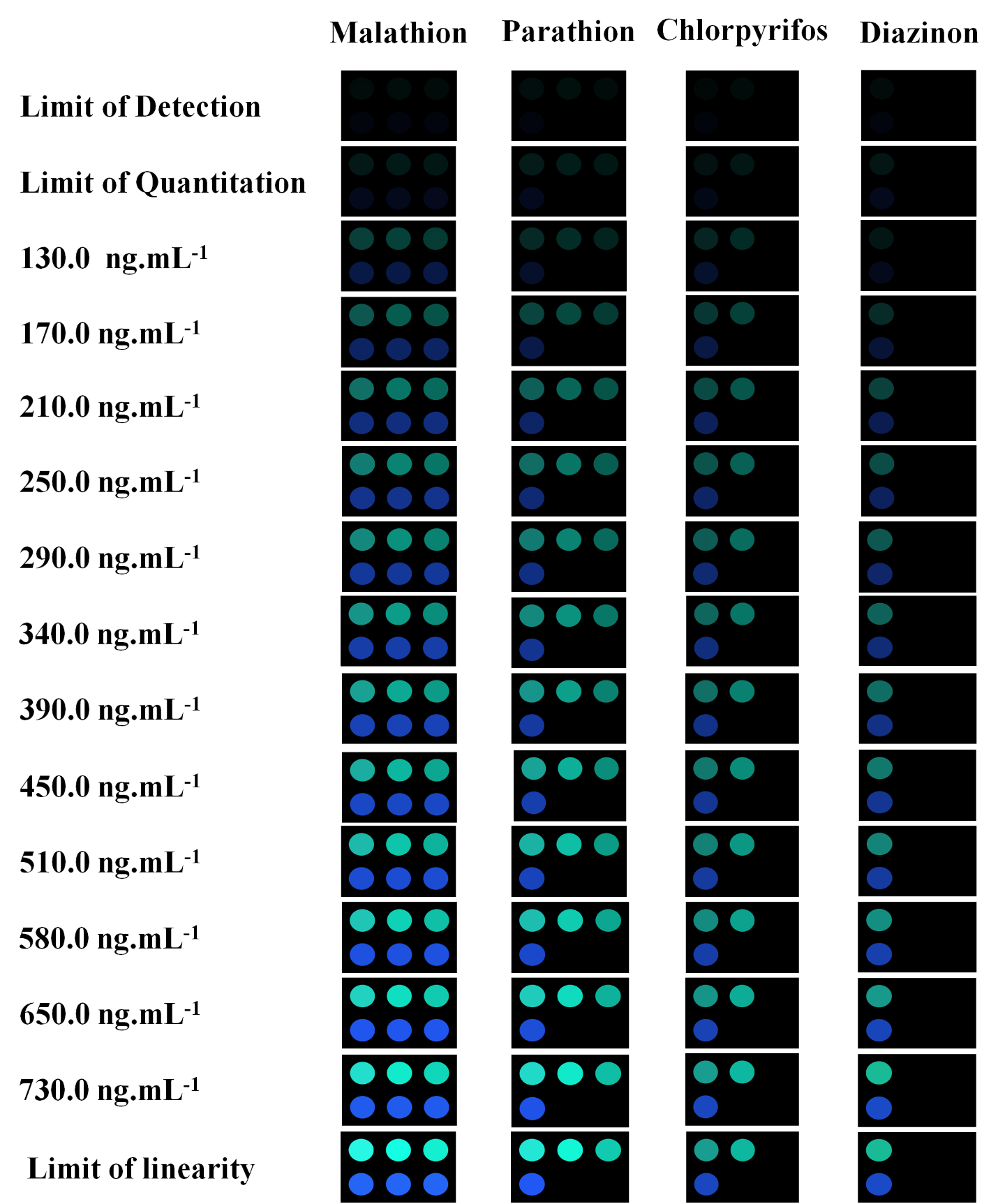


**Figure 13.** The colorimetric difference maps for studied pesticides at different concentrations. Each sensing element was prepared by mixing 0.5 µL of borate buffer (0.1 M) with 0.4 µL of a certain NPs and 0.1 µL of deionized water. The pH of mixture was adjusted at 9.0.


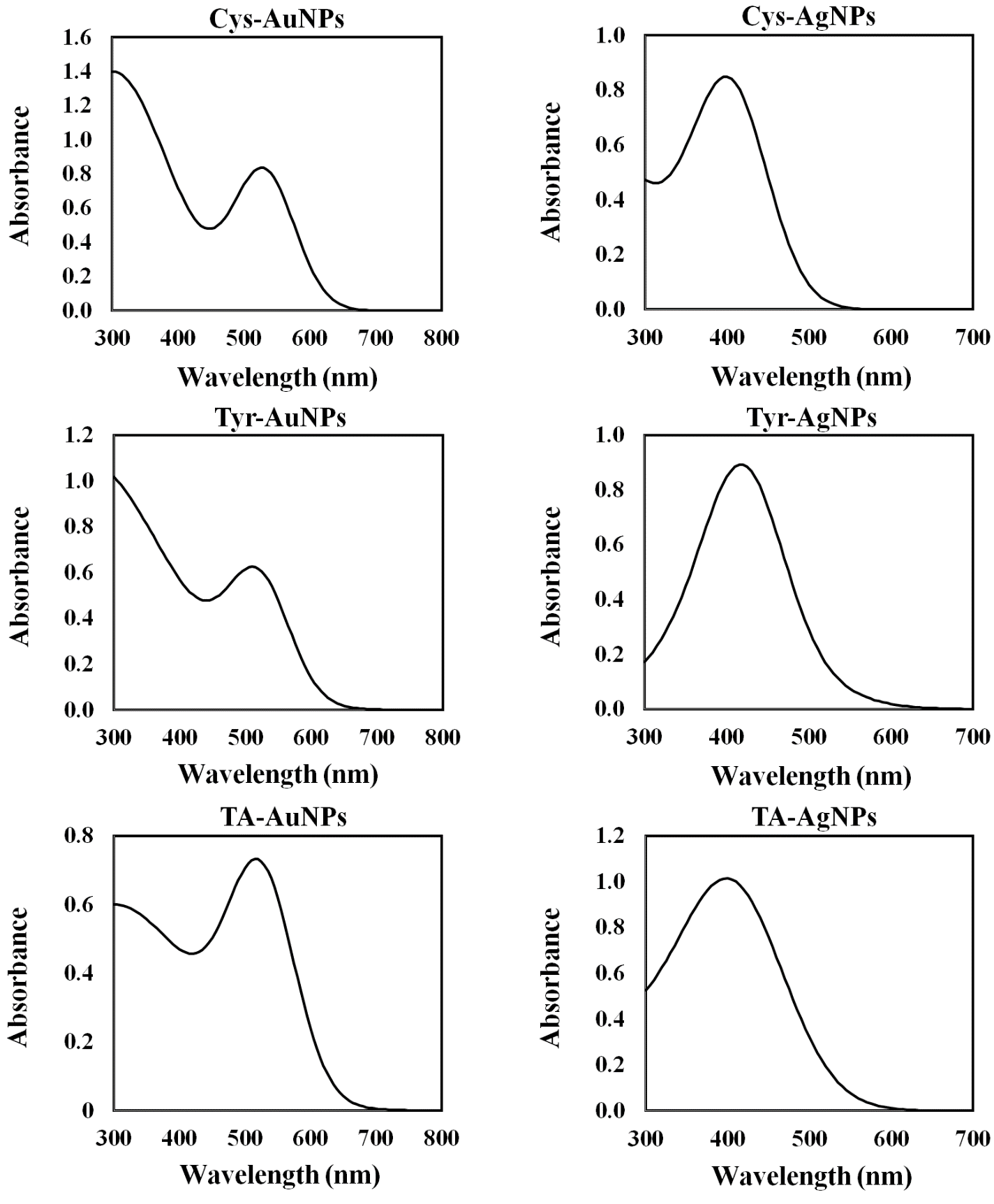


**Figure S14.** Absorption spectra of synthesized NPs obtained by UV-Vis spectrophotometer.


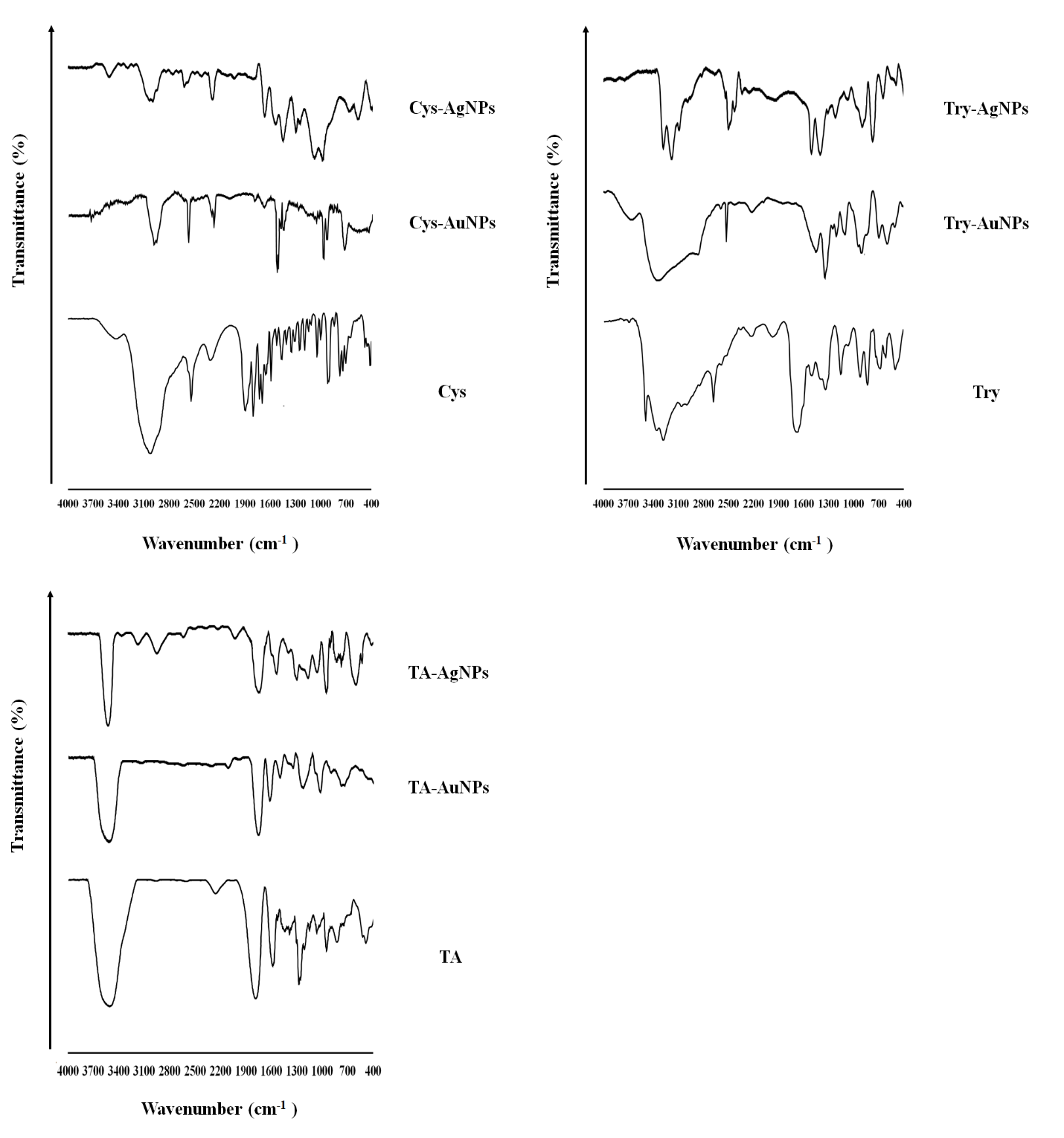


**Figure S15.** FT-IR spectra of synthesized NPs and their capping agents.


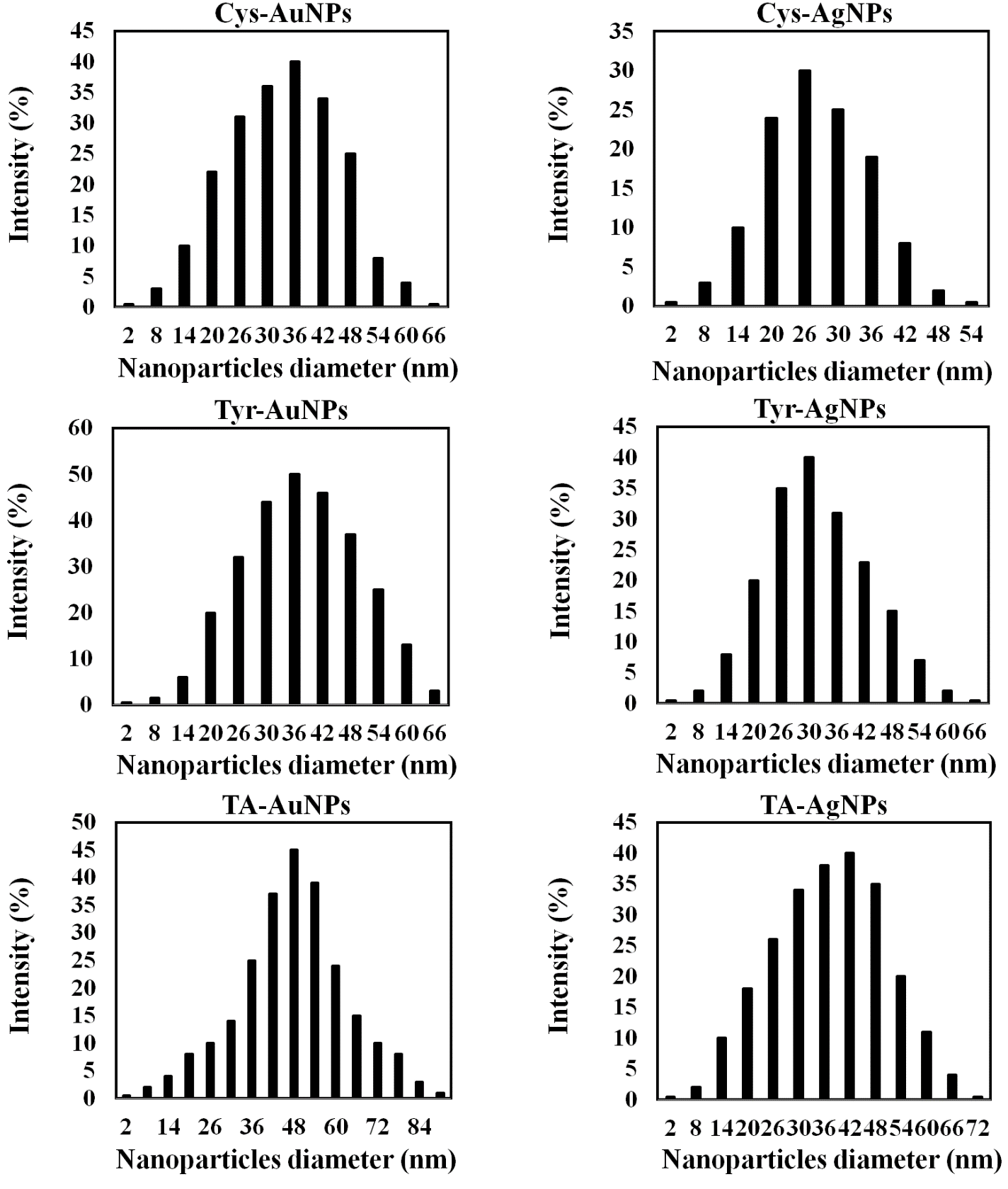


**Figure S16.** Size distribution of synthesized NPs obtained by dynamic light scattering (DLS).


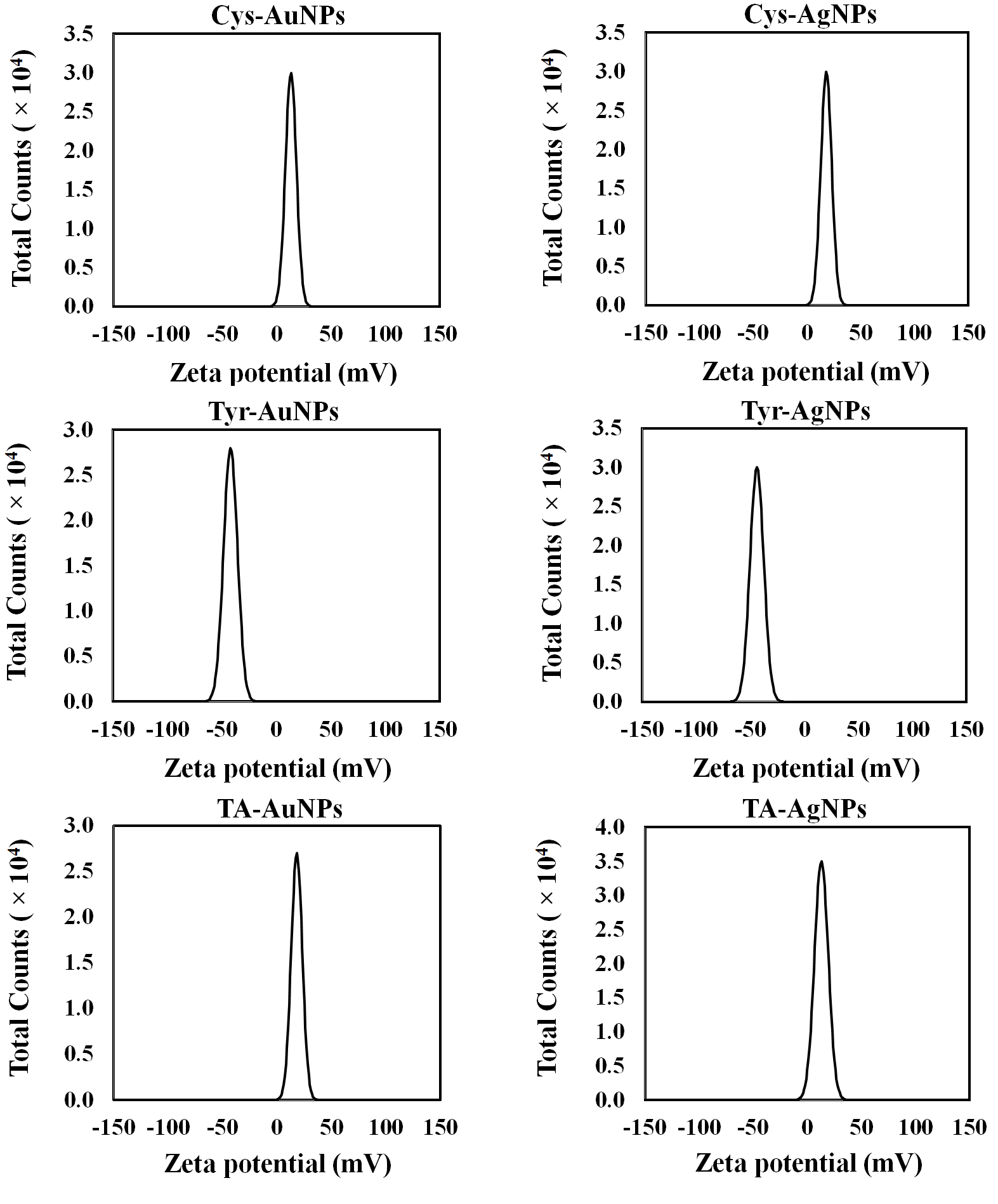


**Figure S17.** Electrical charge on the surface of synthesized NPs.

| **Table S1.** The values of gold and silver elements in the structure of synthesized NPs. the elemental analysis was performed by EDX. | | |
| --- | --- | --- |
| **Type of Nanoparticles** | **Amount of element (%)** | |
|  | **Ag** | **Au** |
| Cys capped AuNPs | - | 21.6 |
| Tyr capped AuNPs | - | 21.8 |
| TA capped AuNPs | - | 23.2 |
| Cys capped AgNPs | 35.3 | - |
| Tyr capped AgNPs | 30.7 | - |
| TA capped AgNPs | 33.4 | - |

**Table S2.** Evaluation of the repeatability of detection zone fabrication.

| **Type of NPs** | **RGB values** | **Number of sensor** | | | | | **RSD %** |
| --- | --- | --- | --- | --- | --- | --- | --- |
|  |  | **S1** | **S2** | **S3** | **S4** | **S5** |  |
| Cys capped AuNPs | R | 225 | 226 | 224 | 225 | 224 | 0.37 |
|  | G | 140 | 142 | 138 | 140 | 141 | 1.06 |
|  | B | 145 | 144 | 144 | 145 | 146 | 0.57 |
| Tyr capped AuNPs | R | 221 | 221 | 223 | 220 | 221 | 0.49 |
|  | G | 129 | 128 | 129 | 129 | 131 | 0.85 |
|  | B | 139 | 142 | 140 | 138 | 139 | 1.09 |
| TA capped AuNPs | R | 219 | 217 | 221 | 219 | 221 | 0.76 |
|  | G | 113 | 113 | 110 | 111 | 113 | 1.26 |
|  | B | 128 | 130 | 130 | 127 | 128 | 1.04 |
| Cys capped AgNPs | R | 226 | 227 | 225 | 225 | 226 | 0.37 |
|  | G | 211 | 208 | 210 | 211 | 210 | 0.58 |
|  | B | 173 | 174 | 176 | 173 | 173 | 0.75 |
| Tyr capped AgNPs | R | 228 | 230 | 226 | 227 | 228 | 0.65 |
|  | G | 208 | 208 | 209 | 208 | 209 | 0.26 |
|  | B | 155 | 158 | 154 | 156 | 155 | 0.97 |
| TA capped AgNPs | R | 232 | 231 | 229 | 230 | 232 | 0.56 |
|  | G | 207 | 207 | 210 | 209 | 207 | 0.68 |
|  | B | 148 | 149 | 147 | 148 | 149 | 0.56 |

**Table S3.** The effect of possible interferences on the assay responses in the presence of pesticides with the concentration of 450.0 ng.mL^-1^.

| **Interferences** | **Tolerance limit^1^**  **( [Foreign species]/[Studied analytes])** |
| --- | --- |
| Ethanol, Methanol, 1-Hexanol, Hexanal, Heptanal, Benzaldehyde, Benzene, Toluene, *p*-Xylene, heptane, hexane, heptane, Dimethylphenylphosphine, Carbaryl, Pirimicarb, Carbofuran, amylamine, Benzylamine, Pyridine, Aniline, Isobutyric acid | 100 |
| Acetic acid, Phosphoric acid, Dichlorvos, Trichlorfon | 50 |
| Paraoxon, Triethylamine, Ammonia | 25 |
| ^1^ The tolerance limit is a criteria determined as the concentration of interference which is shown the relative error less than ±5% in the analyte measurements. | |

**Table S4.** Evaluation of the reproducibility of assay responses to four studied pesticides.

| **Analyte** | **Euclidean Norm for** | | | | | | | | | | **RSD (%)** |
| --- | --- | --- | --- | --- | --- | --- | --- | --- | --- | --- | --- |
|  | **S 1** | **S 2** | **S 3** | **S 4** | **S 5** | **S 6** | **S 7** | **S 8** | **S 9** | **S 10** |  |
| **Malathion** | 544 | 520 | 529 | 568 | 602 | 531 | 579 | 553 | 540 | 583 | 4.87 |
| **Parathion** | 417 | 388 | 441 | 465 | 425 | 403 | 397 | 435 | 426 | 448 | 5.65 |
| **Chlorpyrifos** | 293 | 271 | 305 | 320 | 284 | 286 | 298 | 315 | 310 | 289 | 5.18 |
| **Diazinon** | 227 | 240 | 241 | 207 | 209 | 235 | 239 | 217 | 203 | 243 | 6.95 |

**Table S5.** Guideline for preparation of each mixture of studied pesticides in the both training and prediction set.

| **Set** | **Experiment** | **Malathion**  **(ng.mL^-1^)** | **Parathion (ng.mL^-1^)** | **Chlorpyrifos**  **(ng.mL^-1^)** | **Diazinon**  **(ng.mL^-1^)** |
| --- | --- | --- | --- | --- | --- |
| **Training set** | 1 | 400 | 400 | 400 | 400 |
|  | 2 | 400 | 150 | 150 | 650 |
|  | 3 | 150 | 150 | 650 | 275 |
|  | 4 | 150 | 650 | 275 | 650 |
|  | 5 | 650 | 275 | 650 | 400 |
|  | 6 | 275 | 650 | 400 | 275 |
|  | 7 | 650 | 400 | 275 | 275 |
|  | 8 | 400 | 275 | 275 | 525 |
|  | 9 | 275 | 275 | 525 | 650 |
|  | 10 | 275 | 525 | 650 | 525 |
|  | 11 | 525 | 650 | 525 | 400 |
|  | 12 | 650 | 525 | 400 | 650 |
|  | 13 | 525 | 400 | 650 | 650 |
|  | 14 | 400 | 650 | 650 | 150 |
|  | 15 | 650 | 650 | 150 | 525 |
|  | 16 | 650 | 150 | 525 | 150 |
|  | 17 | 150 | 525 | 150 | 400 |
|  | 18 | 275 | 150 | 400 | 525 |
|  | 19 | 150 | 400 | 525 | 525 |
|  | 20 | 400 | 525 | 525 | 275 |
| **Prediction set** | 1 | 525 | 525 | 275 | 150 |
|  | 2 | 525 | 275 | 150 | 275 |
|  | 3 | 275 | 150 | 275 | 400 |
|  | 4 | 150 | 275 | 400 | 150 |
|  | 5 | 275 | 400 | 150 | 150 |

**Table S6.** Comparison between the analytical ability of the proposed PAD and some reported E-noses for detection of pesticides

| **Type of Sensor** | **Number of Sensor** | **Type of Pesticide** | **Classification analysis** | **Detection limit** | **Sample media** | **Ref.** |
| --- | --- | --- | --- | --- | --- | --- |
| Electrochemical biosensors | 1 | Paraoxon | - | 12.0 µg.mL^-1^ | Air | ^9^ |
| Electrochemical biosensors | 1 | Malathion | - | 12.0 ng.mL^-1^ | - | ^10^ |
| Fluorescent probe | 1 | Diethyl  chlorophosphate | - | 2.6  ng.mL^-1^ | - | ^11^ |
| Commercial gas sensor | 16 | Chlorpyrifos | - | - | - | ^12^ |
| Figaro sensors | 7 | Profenofos | PCA (100 %) and FCM | 6.0 µg.mL^-1^ | Vegetable | ^13^ |
| metal oxide | 16 | Organochlorine | PCA (99 %) and ANN | - | Fruit | ^14^ |
| Dyes | 16 | Oxon organophosphate | PCA (61 %) and HCA (100 %) | 24.0 µg.mL^-1^ | - | ^15^ |
| Nanoparticles | 6 | Thion organophosphate | PCA (100 %) and HCA (100 %) | 58.0 ng.mL^-1^ | Air | This study |

**References**

1. Jv, Y., Li, B. & Cao, R. Positively-charged gold nanoparticles as peroxidiase mimic and their application in hydrogen peroxide and glucose detection. *Chem. Commun.* **46,** 8017–8019 (2010).

2. Dubey, K. *et al.* Tyrosine- and tryptophan-coated gold nanoparticles inhibit amyloid aggregation of insulin. *Amino Acids* **47,** 2551–2560 (2015).

3. Aswathy Aromal, S. & Philip, D. Facile one-pot synthesis of gold nanoparticles using tannic acid and its application in catalysis. *Phys. E Low-Dimensional Syst. Nanostructures* **44,** 1692–1696 (2012).

4. Wang, L. *et al.* Facile, green and clean one-step synthesis of carbon dots from wool: Application as a sensor for glyphosate detection based on the inner filter effect. *Talanta* **160,** 268–275 (2016).

5. Selvakannan, P. *et al.* Probing the effect of charge transfer enhancement in off resonance mode SERS via conjugation of the probe dye between silver nanoparticles and metal substrates. *Phys. Chem. Chem. Phys.* **15,** 12920–12929 (2013).

6. Alam, M. F., Laskar, A. A., Ahmed, S., Shaida, M. A. & Younus, H. Colorimetric method for the detection of melamine using in-situ formed silver nanoparticles via tannic acid. *Spectrochim. Acta - Part A Mol. Biomol. Spectrosc.* **183,** 17–22 (2017).

7. Tavakkoli Yaraki, M. *et al.* Synthesis and optical properties of cysteamine-capped ZnS quantum dots for aflatoxin quantification. *J. Alloys Compd.* **690,** 749–758 (2017).

8. Manikkaraja, C. *et al.* A novel method to detect bovine sex pheromones using L-tyrosine-capped silver nanoparticles: Special reference to nanosensor based estrus detection. *J. Photochem. Photobiol. B Biol.* **203,** (2020).

9. Mishra, R. K. *et al.* Detection of vapor-phase organophosphate threats using wearable conformable integrated epidermal and textile wireless biosensor systems. *Biosens. Bioelectron.* **101,** 227–234 (2018).

10. Baker, P. A., Goltz, M. N., Schrand, A. M., Yoon, D. Y. & Kim, D. S. Organophosphate vapor detection on gold electrodes using peptide nanotubes. *Biosens. Bioelectron.* **61,** 119–123 (2014).

11. Yao, J. *et al.* Concise and Efficient Fluorescent Probe via an Intromolecular Charge Transfer for the Chemical Warfare Agent Mimic Diethylchlorophosphate Vapor Detection. *Anal. Chem.* **88,** 2497–2501 (2016).

12. Marco, F. L. *et al.* Electronic nose for pesticides detection: A first realization. *4th IEEE Int. Work. Metrol. AeroSpace, Metroaerosp. 2017 - Proc.* 403–407 (2017). doi:10.1109/MetroAeroSpace.2017.7999606

13. Tan, S. L., Teo, H. S. & García-Guzmán, J. E-nose screening of pesticide residue on chilli and double-checked analysis through different data-recognition algorithms. *Proc. - 2010 IEEE Electron. Robot. Automot. Mech. Conf. CERMA 2010* 592–596 (2010). doi:10.1109/CERMA.2010.123

14. Ortiz, J. E., Gualdron, O. & Duran, C. M. Detection of pesticide in the vesca fregaria through an electronic nose. *CHILECON 2015 - 2015 IEEE Chil. Conf. Electr. Electron. Eng. Inf. Commun. Technol. Proc. IEEE Chilecon 2015* 679–683 (2016). doi:10.1109/Chilecon.2015.7404643

15. Chulvi, K. *et al.* Discrimination of nerve gases mimics and other organophosphorous derivatives in gas phase using a colorimetric probe array. *Chem. Commun.* **48,** 10105–10107 (2012).
